# Expansion microscopy using a single anchor molecule for high-yield multiplexed imaging of proteins and RNAs

**DOI:** 10.1371/journal.pone.0291506

**Published:** 2023-09-20

**Authors:** Yi Cui, Gaojie Yang, Daniel R. Goodwin, Ciara H. O’Flanagan, Anubhav Sinha, Chi Zhang, Kristina E. Kitko, Tay Won Shin, Demian Park, Samuel Aparicio, CRUK IMAXT Grand Challenge Consortium, Edward S. Boyden

**Affiliations:** 1 McGovern Institute, Massachusetts Institute of Technology (MIT), Cambridge, Massachusetts, United States of America; 2 Media Arts & Sciences, MIT, Cambridge, Massachusetts, United States of America; 3 Department of Molecular Oncology, BC Cancer, Vancouver, British Columbia, Canada; 4 Harvard-MIT Program in Health Sciences and Technology, MIT, Cambridge, Massachusetts, United States of America; 5 Department of Pathology and Laboratory Medicine, University of British Columbia, Vancouver, British Columbia, Canada; 6 Department of Biological Engineering, MIT, Cambridge, Massachusetts, United States of America; 7 Department of Brain and Cognitive Sciences, MIT, Cambridge, Massachusetts, United States of America; 8 Koch Institute for Cancer Research, MIT, Cambridge, Massachusetts, United States of America; 9 Howard Hughes Medical Institute, MIT, Cambridge, Massachusetts, United States of America; Brandeis University, UNITED STATES

## Abstract

Expansion microscopy (ExM), by physically enlarging specimens in an isotropic fashion, enables nanoimaging on standard light microscopes. Key to existing ExM protocols is the equipping of different kinds of molecules, with different kinds of anchoring moieties, so they can all be pulled apart from each other by polymer swelling. Here we present a multifunctional anchor, an acrylate epoxide, that enables proteins and RNAs to be equipped with anchors in a single experimental step. This reagent simplifies ExM protocols and reduces cost (by 2-10-fold for a typical multiplexed ExM experiment) compared to previous strategies for equipping RNAs with anchors. We show that this united ExM (uniExM) protocol can be used to preserve and visualize RNA transcripts, proteins in biologically relevant ultrastructures, and sets of RNA transcripts in patient-derived xenograft (PDX) cancer tissues and may support the visualization of other kinds of biomolecular species as well. uniExM may find many uses in the simple, multimodal nanoscale analysis of cells and tissues.

## Introduction

**Fig 1:**
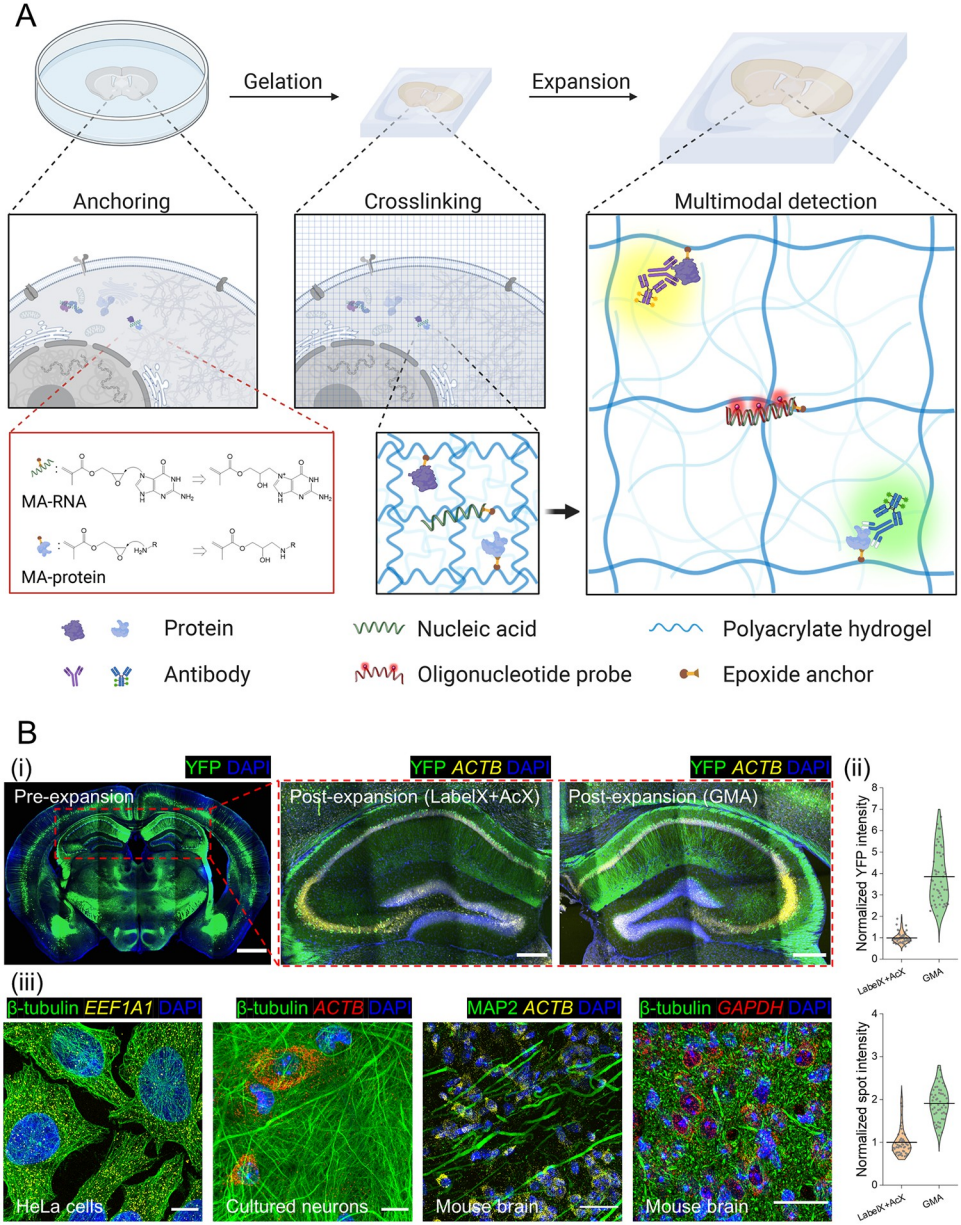
An epoxide anchor enables expansion of proteins and RNAs away from each other. **(A)** In a standard ExM experiment, target biomolecules (*e*.*g*., proteins and nucleic acids, or labels attached to them) in a biological specimen are covalently bound to anchoring molecules bearing vinyl groups (here, an epoxide such as glycidyl methacrylate (GMA), turning biomolecules to methacrylate (MA) forms, *e*.*g*., MA-RNA and MA-protein) that can be crosslinked to a swellable polyacrylate hydrogel synthesized throughout the specimen (“Gelation”). After tissue softening with denaturation and/or proteolysis, the sample can be isotropically expanded upon dialysis with low osmolarity solutions (*e*.*g*., distilled water), during which the anchored biomolecules are pulled apart (“Expansion”). Target-specific detection can be performed (*e*.*g*., antibody staining for proteins and oligonucleotide probe hybridization for RNAs) to realize nanoimaging on conventional microscopes. **(B)** Using the epoxide anchor GMA, simultaneous detection of nucleic acids and proteins was demonstrated across different sample types. In panel **(i)**, a 50 μm thick coronal section of mouse brain tissue expressing Thy1-YFP was cut in half, one part anchored with 0.01% (w/v) LabelX plus 0.005% (w/v) AcX and the other with 0.1% (w/v) GMA. The samples were then subjected to gelation and proK based digestion. HCR-FISH targeting *ACTB* mRNAs was performed post-expansion and the FISH signals were quantified together with the retained YFP signals. Scale bars (in pre-expansion units): 1000 μm (whole brain); 250 μm (zoomed-in hippocampal view). Linear expansion factor: 4.1. In panel **(ii)**, mean intensities for YFP within cells and HCR-FISH spots in each image were quantified and compared between the LabelX/AcX and GMA processed tissues (data distribution shown in violin plots, with raw data points presented, and mean values highlighted with solid lines; n = 50 images from 3 different slices, 2 mouse brains; two-sample *t*-test was performed with *p* < 10^−20^ for both YFP and HCR-FISH signals). In panel **(iii)**, multimodal detection of proteins and RNAs with uniExM was demonstrated. Antibody staining for proteins was performed pre-expansion and the samples were anchored with 0.04% (w/v) GMA (for HeLa cells and cultured neurons) or 0.1% (w/v) GMA (for mouse brain slices). After gelation and expansion, HCR-FISH targeting specific RNA targets was performed post-expansion. In the figure captions, capitalized italic fonts represent the RNA target names (*ACTB*, *EEF1A1*, *GAPDH*). The colors in each image correspond to the following fluorescent dyes: blue—DAPI; green—Alexa488; yellow—Alexa546; red—Alexa647. Scale bars (in pre-expansion units): 20 μm. Linear expansion factors: 4.4 for HeLa cells and cultured neurons, 4.1 for mouse brain tissues (prior to re-embedding). All images are shown as maximum z-projection of image stacks (10–15 μm z-depth for cultured HeLa cells and neurons; 50 μm for mouse brain tissues).

Nanoscale imaging enables the analysis of biological systems, such as cells and tissues, at the fundamental length scales of biomolecules and biomolecular interactions, but classically has required expensive equipment and advanced skillsets to perform, *e*.*g*., via super-resolution microscopy [1]. Recently, expansion microscopy (ExM), which physically magnifies biological specimens through a chemical process, thus enabling nanoimaging on conventional microscopes [2–4]. has become popular, with hundreds of experimental papers and preprints exploring a diversity of biological systems with nanoscale precision [5]. ExM protocols comprise several typical steps: first, one or more molecular anchors are introduced to covalently bond with target biomolecules, or labels bound to those molecules (*e*.*g*., endogenous proteins or nucleic acids, or fluorescent probes bound to them); second, acrylate monomers are infused and then polymerized into a swellable polyacrylate hydrogel network that also binds to the anchors; third, the resultant specimen-hydrogel composite is subjected to denaturation or enzymatic digestion to free up inter/intra-molecular connections (*e*.*g*., fixative crosslinks), or even to dissolve molecules no longer needed for visualization; finally, the sample is isotropically expanded (typically ~4.5× in the most commonly used protocols [6]) upon dialysis with an excess of water (Fig 1A). ExM has been successfully demonstrated in a wide range of sample types and given rise to a number of variants tackling specialized purposes, *e*.*g*., higher magnification factors, adaptation to human tissues, decrowding of molecules for better access by labels, and multiplexed molecular imaging [7–26], to name a few, which have been used to study a diversity of topics in virology, molecular biology, neuroscience, cancer biology, and other fields within biology and medicine.

Although most papers performing ExM investigate a single kind of biomolecule, *e*.*g*., proteins, an increasing number of studies are seeking to investigate multiple kinds of molecule, *e*.*g*., both proteins and RNAs [4, 21, 27, 28]. The recently published Magnify protocol yielded images of RNA being visualized in cultured cells, but the RNA yield was not measured, nor compared to earlier methods in terms of quantitative performance; furthermore, dual RNA and protein visualization in the same sample was not performed [29]. Except for this protocol, all other protocols for examining both proteins and RNAs to date have required different anchors—proteins have been bound by molecules that connect amines to a vinyl group (*e*.*g*., through the molecule AcX) [3, 7, 8], whereas RNAs have been bound by molecules that alkylate guanines (*e*.*g*., through the molecule LabelX) [4, 27] (schematics in S1 Fig in S1 File). The RNA anchors are made by mixing off-the-shelf chemicals overnight; the RNA anchors are sometimes applied to the specimen in a separate step from the protein anchors, adding time and complexity to the procedure. The need for in-house synthesis adds a potential uncertainty to the overall process, since individuals may not conduct the reaction under identical conditions in all groups, resulting in non-controlled final yield. The cost of the RNA-binding reagents is also high—LabelX and MelphaX (AcX reacted with Label-IT amine and Melphalan, respectively), the two molecules used to date, cost $7,500 and $70 per mg, respectively, as of late 2022, resulting in overall experimental costs of $180 and $40, respectively, for a typical sample (*e*.*g*., a 50–100 μm thick full-width mouse brain slice. Here we introduce, and validate, an epoxide-based anchoring strategy that enables proteins and RNAs to be anchored to the hydrogel in a single step, in an inexpensive fashion, without requiring any end-user synthesis. We demonstrated the versatility of this united ExM (uniExM) protocol in the analysis of proteins and RNAs, as well as in multiplexed settings such as that of expansion sequencing (ExSeq) [21], suggesting the utility of epoxide anchoring in a diversity of high-resolution spatial biology studies.

## Materials and methods

### Cell culture and mouse brain tissues

HeLa cells were cultured in DMEM medium supplemented with 10% FBS and 1% penicillin-streptomycin antibiotics. Once the cells reached 70–80% confluence, they were fixed with either 4% paraformaldehyde (PFA) or 3% PFA/0.1% glutaraldehyde (GA, for better preservation of intracellular fine structures including β-tubulin and βII-spectrin in Figs 1B iii, 2A and 3, S7, S13B Figs in S1 File), followed by residual aldehyde quenching with 0.1% sodium borohydride and 100 mM glycine. For RNA detection (ExFISH and ExSeq), samples were permeabilized and stored with 70% ethanol at 4°C overnight or up to 4 weeks.

**Fig 2:**
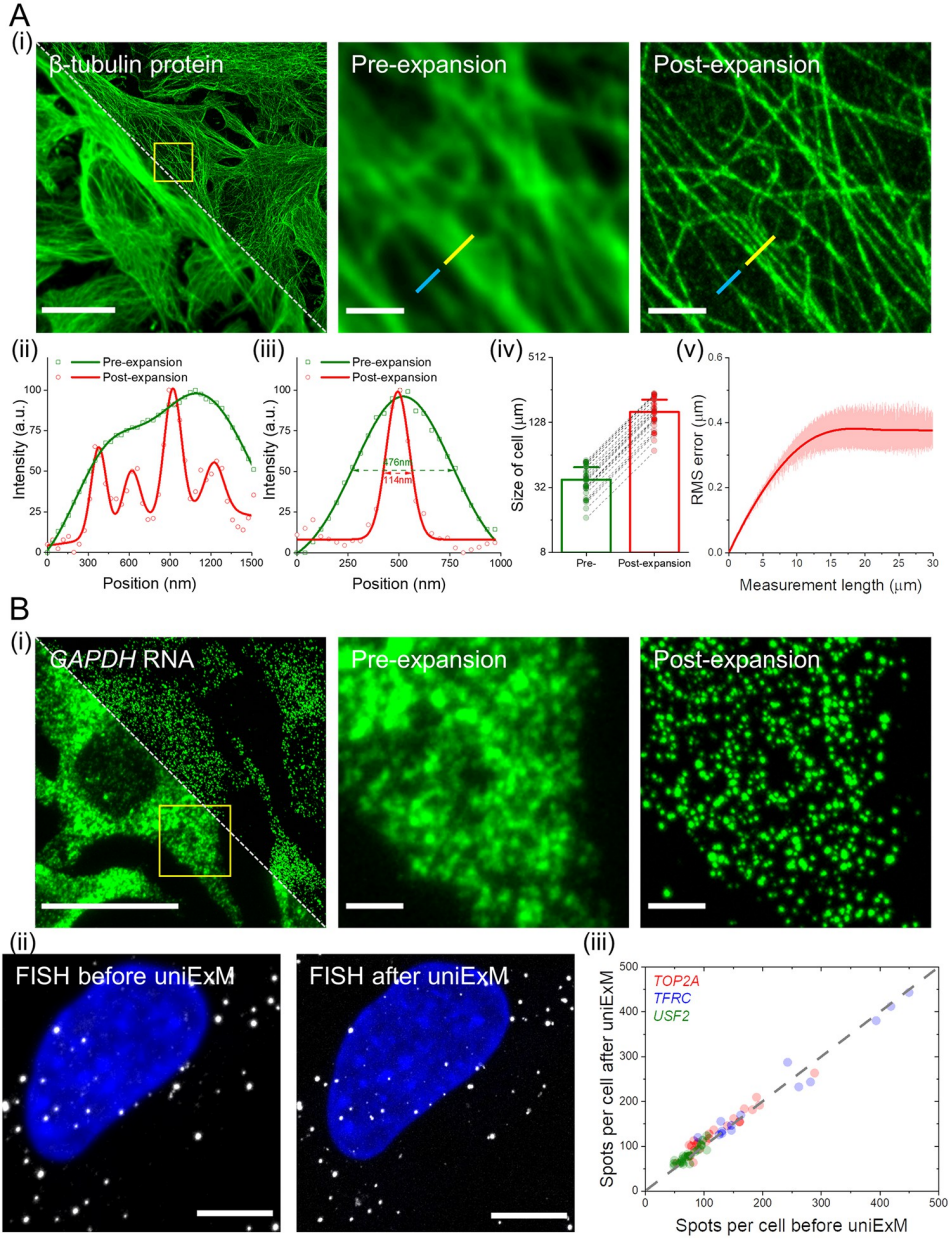
Characterization of GMA-based uniExM for protein and RNA retention. **(A)** uniExM improves imaging resolution and achieves homogenous expansion. **(i)** Representative images of HeLa cells stained with β-tubulin antibody (pre-expansion staining) are shown. Upon expansion, resolution improvement, expansion factor and distortion were evaluated. Left image: pre-expansion (lower left half) and post-expansion (upper right half) fields of view of the same specimen, where the white diagonal dashed line delineates the boundary between the two images. Middle and right images: zoomed-in view of the region highlighted by the yellow square in the left image. Scale bars (in pre-expansion units): 20 μm (left image), 2 μm (middle and right images). Panels **(ii)** and **(iii)** plot the cross-section intensity profiles along the yellow and blue lines, respectively, in the zoomed-in images of panel **(i)**. The raw intensity values (shapes) were fitted with multi-peak Gaussian functions (solid lines). The values presented in panel **(iii)** are FWHM (full width at half maximum) of the fitted Gaussian functions. In panel **(iv)**, long axes of the same cell were measured before and after expansion to calculate the expansion factor. The linear expansion factor was determined to be 4.2 in this demonstration (n = 30 cells from 2 different batches of culture; mean + standard deviation was presented in bar chart with raw measurements shown as individual points). In panel **(v)**, RMS length measurement error was quantified by benchmarking post-expansion confocal images against pre-expansion super-resolution SoRa images of microtubule staining in HeLa cells (red line, mean value; shaded area, standard deviation; n = 5 samples). **(B)** uniExM for RNA detection and quantification. **(i)** GMA-based expansion helps de-crowd densely packed mRNAs and better resolve single transcripts of the highly expressed *GAPDH* gene in HeLa cells. Left image: pre-expansion (lower left half) and post-expansion (upper right half) images of the same specimen, where the white diagonal dashed line delineates the boundary between these two images. Middle and right images: zoomed-in view of the region highlighted by the yellow square in the left image. Scale bars (in pre-expansion units): 20 μm (left image), 2 μm (middle and right images). **(ii)** GMA-based uniExM effectively preserves RNA information during the expansion process. HCR-FISH targeting specific genes was performed before and after expansion. Left image: a representative image of HCR-FISH for the *USF2* gene in HeLa cells. Number of transcripts per cell was counted, and then FISH probes were stripped off with concentrated formamide and heating. Right image: The same sample was subjected to uniExM, after which HCR-FISH targeting the same gene was performed and quantified. Scale bars (in pre-expansion units): 5 μm. **(iii)** Three genes—*TOP2A*, *TFRC and USF2* (with the expression level ranging from ~50 to ~500 transcripts per cell)—were chosen to evaluate the RNA anchoring efficiency by GMA in uniExM. Spots/transcripts per cell counted before and after GMA anchoring were fit by linear regression, with an R-squared value of 0.9736, indicating nearly 100% RNA retention (each point in the scatter plot represents one measurement from a single cell; n = 60 cells collected from 3 culture batches).

**Fig 3:**
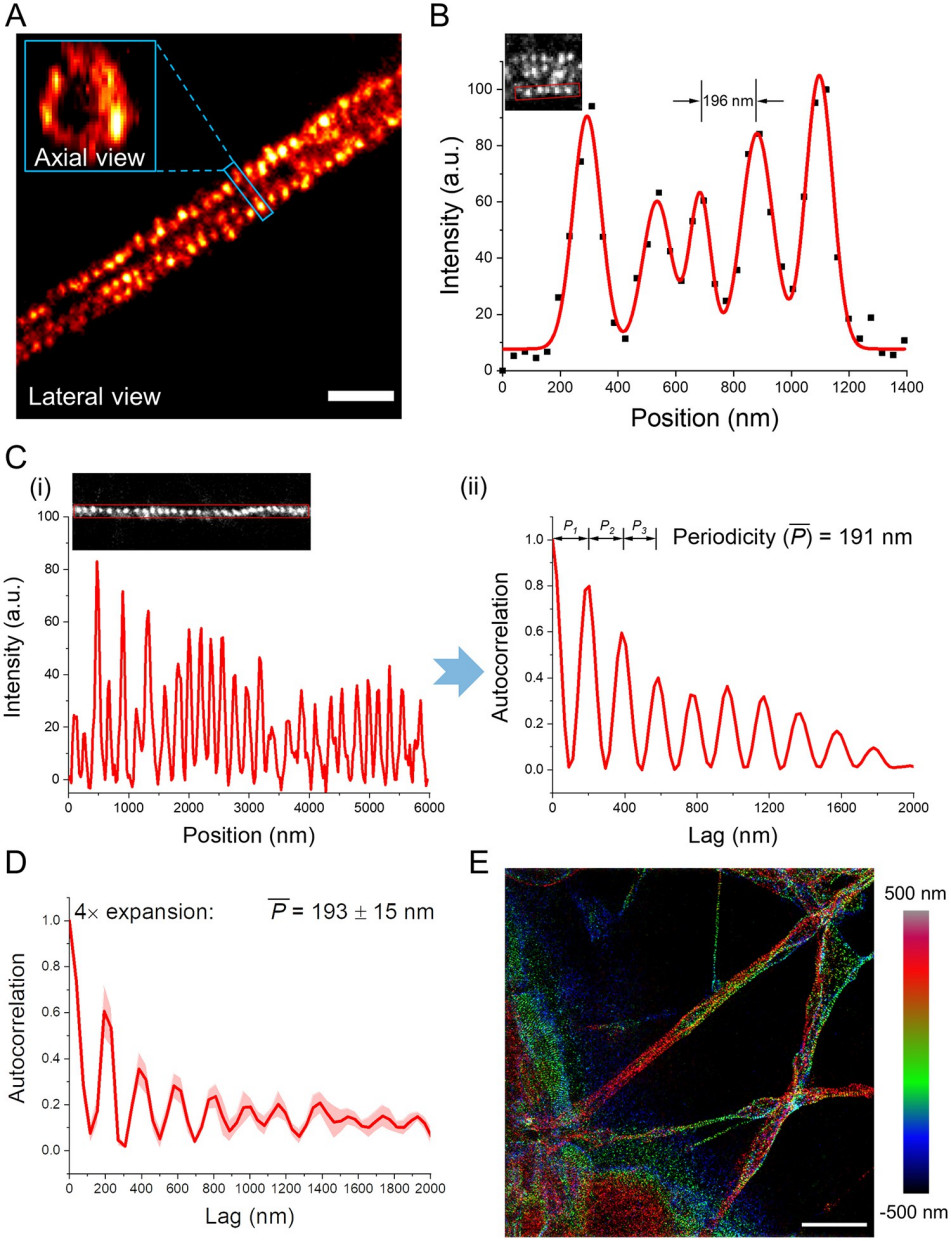
Preservation of protein ultrastructural organization by GMA in uniExM. **(A)** Antibody staining against βII-spectrin in mouse hippocampal neurons was performed with 4× expansion (linear expansion factor ~4.2). One segment of axon showing periodic, punctate signals is presented. In the zoomed-in inset, the axial view of βII-spectrin is shown, displaying a ring structure. Scale bar (in pre-expansion units): 1 μm. **(B)** The intensity profile of βII-spectrin clusters along an axon segment (within the red rectangle of the inset image) was plotted and fitted with multi-peak Gaussian functions (black squares: raw data points; red line: fitting function). A consistent distance around 190 nm between two adjacent peaks was noted. **(C)** Antibody staining against βII-spectrin in mouse hippocampal neurons was performed with a 7× expansion protocol modified from the TREx protocol (linear expansion factor ~7.3). **(i)** The intensity profile of over 10 βII-spectrin clusters along an axon segment (within the red rectangle of the inset image) was plotted. **(ii)** Autocorrelation analysis was performed to calculate the periodicity of βII-spectrin clusters across space (*i*.*e*., the similarity between signals as a function of the spatial position lag between them). Based on each fitted autocorrelation function, the first three inter-peak distance values (denoted as *P*_*1*_, *P*_*2*_ and *P*_*3*_) were extracted to calculate the mean periodicity *P̅*. **(D)** Mean autocorrelation function of periodicity analysis using 4× expansion and pre-expansion antibody staining. (Solid lines, mean; shaded areas, standard error of mean. n = 40 measurements from 2 culture batches). **(E)** Post-expansion antibody staining revealed the same periodic distribution pattern of βII-spectrin under 4× expansion. Scale bar (in pre-expansion units): 5 μm. The color code of the image represents z-axis information.

All procedures involving animals were in accordance with the US National Institutes of Health Guide for the Care and Use of Laboratory Animals and approved by the Massachusetts Institute of Technology Committee on Animal Care. Primary neurons were dissected from brains isolated from euthanized newborn Swiss Webster mice and about 1,000 hippocampal neurons were seeded onto a 12 mm #1.5 coverslip. The neurons were cultured with MEM medium containing 33 mM glucose, 0.01% transferrin, 10 mM HEPES, 2 mM Glutagro, 0.13% insulin, 2% B27 supplement, and 7.5% heat-inactivated FBS at 37°C in a humid incubator supplemented with 5% CO_2_ for 2 weeks and then fixed for subsequent uses.

For mouse brain tissues, three seven-week-old mice were terminally anesthetized with isoflurane, followed by transcardial perfusion with PBS and ice cold 4% PFA. Then the brain was dissected out and placed in 4% PFA for 12–16 hours. 50 μm slices were prepared on a vibratome (Leica VT1000s) and then stored at 4°C in PBS (for lipid co-detection in S13 Fig in S1 File) or 70% ethanol until use. Animal health and behavior were fully assessed twice a week before euthanasia.

### Patient-derived xenografts (PDX)

Patient tumor sample xeno-transplanted was acquired in 2011 according to procedures approved by the Ethics Committees at the University of British Columbia. Sample from breast cancer patients undergoing diagnostic biopsy or surgery were collected under protocol H06-00289 (BCCA-TTR-BREAST). Written informed consent was obtained and documented by UBC BC Cancer Research Ethics Board electronically and or in writing. All data were fully anonymized. Tumor fragments were finely chopped and mechanically disaggregated for one minute using a Stomacher 80 Biomaster (Seward Limited, Worthing UK) in 1 mL cold DMEM/F-12 with Glucose, L-Glutamine and HEPES (Lonza 12-719F). 200 μL of medium containing cells/organoids from the suspension was used for transplantation per mouse. Tumors were transplanted in mice as previously described in accordance with SOP BCCRC 009 [30] in 2020. Female NOD/SCID/IL2Rγ −/− (NSG) and NOD/Rag1−/−Il2Rγ −/− (NRG) mice were bred and housed at the Animal Resource Centre at the British Columbia (BC) Cancer Research Centre. Disaggregated cells and organoids were resuspended in 150–200 μl of a 1:1 v/v mixture of cold DMEM/F12: Matrigel (BD Biosciences, San Jose, CA, USA). 8–12-week-old mice were anesthetized with isoflurane and the suspension was transplanted under the skin on the left flank using a 1 mL syringe and 21-gauge needle. One xenograft-bearing mouse was used; it was euthanized for tissue collection when the size of tumor approached 1 mL in volume. The animal care committee and animal welfare and ethical review committee, the University of British Columbia (UBC), approved all experimental procedures. Animal health and behavior were monitored daily, and fully assessed twice a week before euthanasia.

### Anchoring, gelation, homogenization and expansion

Fixed cells and tissue slices were first pre-incubated with 100 mM sodium bicarbonate (pH = 8.5, DNase/RNase-free) twice for 15 min each, and incubated in GMA in 100 mM sodium bicarbonate for 3 h at room temperature or 37°C, dependent on target and sample types (detailed anchoring conditions are provided in S4 Table in S1 File). Of particular note, the solubility of GMA is about 3% in most aqueous solutions and so the anchoring buffer has to be vigorously vortexed after addition of GMA. According to the safety data sheet of GMA, handling of undiluted GMA needs to be done in a fume hood with sufficient ventilation. For experiments using cultured cells, 0.04% (w/v) GMA was used for anchoring, while 0.1% (w/v) GMA was used for tissue samples. For ease of use, the GMA reaction can be left at room temperature overnight without adverse effects. After the anchoring reaction, samples were washed with sterile PBS or DPBS three times (for samples using >0.2% GMA in the concentration optimization experiments of S5 Fig in S1 File, they were washed with 70% ethanol to remove unreacted GMA before washing with DPBS). Then, standard ExM steps including gelation, digestion and expansion were conducted.

Briefly, for gelation, the monomer solution—StockX—was prepared as developed inpublished protocols [6]: 8.6% (w/v) sodium acrylate (SA), 2.5% (w/v) acrylamide (AA), 0.15% (w/v) N,N’-methylenebisacrylamide (Bis), 2 M sodium chloride (NaCl), 1× PBS. Then the gelation solution was prepared by mixing StockX with 0.5% (w/v) 4-Hydroxy-TEMPO (4-HT) stock solution (required for tissue samples), 10% (w/v) N,N,N′,N′-Tetramethylethylenediamine (TEMED) stock solution, and 10% (w/v) ammonium persulfate (APS) stock solution at 47:1:1:1 ratio on a 4°C cold block, and diffused into the sample at 4°C for 30 min. #0 coverslips were used as spacers between two slides to make a gelling chamber, to cast the gel with thickness around 100 μm. Next, the gelling chamber containing the tissue with infiltrated gelation solution was transferred to a sealed Tupperware for free-radical initiated polymerization at 37°C (a detailed ExM manual can be found here [31]). For the modified 7× expansion protocol, the following monomer solution was used (adapted from the TREx protocol [26]): 17.5% SA, 5% AA, 0.015% Bis, 2 M NaCl, 1× PBS, and mixed with 10% TEMED and APS at 198:1:1 ratio. To reduce the gel attachment to glass surfaces, the glassware can be briefly rinsed with Sigmacote reagent before use.

After 2 hours, the gelled sample was removed from the chamber, trimmed to the proper size, where only the areas of interest were kept, and then immersed in digestion buffer. Different sample homogenization methods were applied as specified below.

For experiments in Figs 1–3, S4–S6, S10, S11A, S11B Figs in S1 File, the standard proteinase K (proK) based digestion method was performed with the buffer containing 8 U/ml proK, 0.5% (w/v) Triton X-100, 1 mM EDTA, 50 mM Tris-HCl buffer (pH = 8), and 2 M NaCl. The gelled samples were digested at 37°C overnight.

For the comparison experiments of LabelX/AcX and epoxides in preservation of proteins under high heat treatments (S8 Fig in S1 File), two homogenization methods were tested. In the heat-based SDS denaturation, gels were incubated with the denaturation buffer containing 200 mM SDS, 200 mM NaCl, and 50 mM Tris base (pH = 9) at 95°C for 1 hour, followed by incubation at 37°C overnight and washing with 0.2% TritonX-100 in fresh PBS to remove residual SDS before expansion. In the proK based rapid digestion, gels were digested with 8 U/ml proK, 0.8 M guanidine hydrochloride, 0.5% (w/v) Triton X-100, 1 mM EDTA, 50 mM Tris-HCl buffer (pH = 8), and 2 M NaCl at 60°C for 2 hours, followed by incubation with fresh digestion buffer at 37°C overnight before expansion.

For post-expansion antibody staining and experiments involving WGA staining (S3, S11C, S12C, S13 Figs in S1 File), gelled samples were digested with 50 μg/mL (for cells) or 100 μg/mL (for tissues) endoproteinase LysC in 1 mM EDTA, 50 mM Tris-HCl (pH = 8) and 0.1 M NaCl at 37°C overnight (for cells) or 2–3 days (for tissues). For post-expansion antibody staining targeting Thy1-YFP in mouse brain tissues (S3 Fig in S1 File), the heat-based SDS denaturation was applied.

After homogenization, the gelled samples were rinsed 3 times with fresh PBS, followed by expansion with ion-free, ultrapure water (3 × 15 min for cells, 3 × 30 min for tissues). To expand LysC-digested tissues, serial incubation with decreasing PBS (1×, 0.5×, 0.1×, 30 min each) or NaCl solutions (1M, 0.5M, 0.1M, 30 min each) was conducted before expansion with water.

### Pre-expansion antibody staining

To assess the potential sample distortion during the GMA-based uniExM procedure and the improvement on imaging resolution, pre-expansion antibody staining was performed. Primary antibodies against β-tubulin, MAP2, neurofilament, GFP, and βII-spectrin were used to stain predetermined structures in different samples. In brief, samples were fixed with 3% PFA/0.1% GA (for microtubule and spectrin preservation) or 4% PFA, followed by processing with 0.1% sodium borohydride and 100 mM glycine to quench unreacted fixative residuals. MAXblock medium was used for blocking for 1 hour and then 5 μg/mL primary antibody diluted in MAXbinding medium was incubated with the sample at 4°C overnight (or 37°C for 2 hours). Next day, 5 μg/mL fluorescently labelled secondary antibody was used at room temperature for 1 hour. After completely washing out unbound antibodies, samples were proceeded with the anchoring and expansion steps.

### Post-expansion antibody staining

For post-expansion antibody staining, gelled specimens were treated with the LysC-based digestion (in S3E and S7C Figs in S1 File) or heat-based SDS denaturation (in S3 Fig in S1 File), then followed by expansion [3]. Then the samples were incubated with 5 μg/mL primary antibody diluted in MAXbind staining medium at 4°C overnight (or 37°C for 2 hours) and washed with MAXwash medium four times. 5 μg/mL fluorescently labelled secondary antibody was incubated with the sample to develop signals before DAPI counterstaining and expansion.

### Staining for lipids and carbohydrates

R18, FM and BODIPY were all tested for pre- and post-expansion staining. The working concentration for these dyes was chosen to be 10 μg/mL (diluted with fresh PBS). For pre-expansion staining, lipid tags were introduced right before the gelation step in which samples were stained for 1–2 hours at room temperature. Then the samples were anchored with GMA at 4°C overnight, followed by incubation at room temperature for another 3 hours, digestion (proK-based) and expansion. For post-expansion staining, samples were fixed with 3% PFA/0.1% GA, and then anchored with GMA. The samples were digested with proK or LysC (for WGA staining), followed by expansion. After expansion, the samples were stained with antibodies or HCR-FISH first (if at all), and then with lipid tags or WGA-A647 for 1–2 hours at room temperature. The working concentration for WGA-A647 was chosen to be 5 μg/mL. Residual dyes were washed off with 1% Zwittergent in DPBS.

### Expansion fluorescence *in situ* hybridization (ExFISH)

For ExFISH experiments (in Fig 1B, S3, S6, S10B Figs in S1 File) expanded gels were re-embedded in 3% AA-based (plus 0.15% BIS, 5 mM Tris base pH 9, and 0.05% APS/TEMED), non-expandable gel to maintain rigidity. With the re-embedding step, the expansion factor would decrease to ~3.2 compared to the original expansion factor of ~4.2. Two stacked #1.5 coverslips were usually used as the spacers between two glass slides for re-embedding. The HCR-FISH probes and reagents were purchased from Molecular Instruments, Inc. In general, the gel was incubated with hybridization buffer at room temperature for 30 min, and then with 1:500 diluted gene-specific probe (8 nM total final probe concentration) set at 37°C overnight. Next day, the gel was washed with HCR washing buffer at 37°C for 4 × 30 min and with 5× SSCT buffer (5× SSC buffer containing 0.1% Tween 20) at RT for 4× 15 min, followed by incubation with 1:200 diluted, fluorescently labelled HCR hairpin amplifiers at room temperature overnight. Lastly, the gel was washed with 5× SSCT for 4 × 20 min and counterstained with 1 μg/mL DAPI. To characterize RNA capture efficiency by GMA, HCR-FISH against the same genes was performed in the same sample before and after anchoring. Before anchoring, HCR-FISH was done in HeLa cells and then the hybridized probes were removed with 80% formamide. Then, ExFISH after GMA-based uniExM was done with the same sample, where the same cells were imaged in both conditions. Transcripts in single cells were counted using MATLAB scripts as developed before [32, 33].

### Expansion Sequencing (ExSeq)

The detailed protocol for ExSeq was published previously and involves a multi-day procedure [21]. 87 target genes were chosen to differentiate two major cancer clones in the SA501 PDX line (full gene list is provided in S5 Table in S1 File) [34]. In brief, a re-embedded gel was passivated with 2 M ethanolamine, 150 mM EDC and NHS. Then the passivated gel was subjected to targeted ExSeq (tExSeq) or untargeted ExSeq (uExSeq). For tExSeq, padlock probes targeting specific mRNAs (in general, 12–16 probes per gene and 100 nM per padlock probe diluted in 2× SSC containing 20% formamide) were used to hybridize with the sample at 37°C overnight. Then the unhybridized probes were completely washed off with fresh hybridization buffer and the sample was treated with 1.25 U/μL PBCV-1 DNA ligase at 37°C overnight, followed by inactivation at 60°C for 20 min. Next, the successfully ligated padlock probes were rolling circle amplified with 1 U/μL phi29 DNA polymerase at 30°C overnight. As all padlock probes targeting the same gene bear a predetermined barcode, the identity of the mRNA can be read out by commercially available sequencing reagents (*e*.*g*., the Illumina MiSeq kit). In comparison, uExSeq utilizes randomized 8N oligonucleotide probes to hybridize with any potential RNA targets without prior sequence knowledge. After that, reverse transcription was performed *in situ* with 10 U/μL SSIV reverse transcriptase to generate cDNAs containing inosine. The cDNAs were later segmented to proper sizes with endonuclease V and circularized with 3 U/μL CircLigase. Then the target mRNAs were digested away with RNase H. Such circularized cDNAs were subjected to rolling circle amplification and sequencing readout. For detailed working mechanism and protocols of tExSeq and uExSeq, please refer to our previous work [21].

We adapted the sequencing-by-synthesis chemistry for *in situ* 7-base readout using the Illumina MiSeq v3 kit with a modified protocol. To help the registration process, the re-embedded gel sample was adherent to bind-silane (1:250 diluted in 80% ethanol) processed glass surface with the same re-embedding monomer solution containing 1:100 diluted TetraSpeck microspheres. Before sequencing, the sample was first treated with 400 U/mL terminal transferase and 50 μM ddNTP to block nonspecifically exposed 3’ ends in DNA, and then hybridized with 2.5 μM sequencing primer (5’-TCT CGG GAA CGC TGA AGA CGG C-3’) in 4× SSC at 37°C for 1 hour. After 3 × 10 min washing with fresh 4× SSC, the sample was incubated with the PR2 incorporation buffer (part of the MiSeq kit) for 2 × 15 min. Then the sample was pre-incubated with 0.5× incorporation mix buffer (IMT of the MiSeq kit) supplemented with 1× Taq polymerase buffer and 2.5 mM magnesium chloride at RT for 2 × 15 min. Then the sample was incubated with 0.5× IMT at 50°C for 10 min for one base elongation. After the elongation reaction, the sample was washed with PR2 containing 2% Zwittergent at 50°C for 2 × 15 min followed by additional washing with PR2 at RT for 2 × 15 min. Next, the sample was immersed in imaging buffer (SRE of the MiSeq kit) and subjected to imaging (elaborated in the following section). After imaging, the sample was briefly washed with PR2 at RT for 2 × 10 min. Then the sample was incubated with cleavage solution (EMS of the MiSeq kit) at 37°C for 3 × 15 min. Lastly, the sample was washed with PR2 at 37°C for 2 × 15 min and at RT for 2 × 15 min, and then started with the next round of elongation process.

### Data analysis for ExSeq

Data analysis for the sequenced PDX sample followed our established ExSeq processing pipeline (available at: https://github.com/dgoodwin208/ExSeqProcessing). For the 87-gene probe set, a 7-base barcoding strategy with error correction capacity was adopted. Upon microscopic readout, the raw image files were stored in 16-bit HDF5 format and subjected to color correction, registration, segmentation, basecalling and alignment as done in our previous work [21], and then performed manual cell segmentation in 2D according to a max-Z projection of the DAPI staining channel using the VASTLite package (https://lichtman.rc.fas.harvard.edu/vast/). In total, 793,535 unique transcripts were detected for a population of 3,339 cells (with effective lateral resolution ~78 nm and axial resolution of ~160 nm). For gene function annotation we refer to The Human Protein Atlas (https://www.proteinatlas.org) or The Human Gene Database (https://www.genecards.org). The spatial maps of single transcripts or functional gene groups were generated with MATLAB scripts (for coordinates extraction) and ImageJ packages (for visualization).

For single-cell biostatistics analysis we utilized the R toolkit Seurat 4. Before analysis, we further pruned the dataset based on the counts per cell values, where cells with less than 50 counts or more than 3000 counts were filtered out and we ended up with 2,732 cells. Then we normalized the counts per cell by the median value from all the cells and performed a log transformation. To identify cell clusters, we applied both unsupervised and supervised approaches. In unsupervised clustering, PCA suggested a majority of cells could be classified into two groups using the expression of 30 genes automatically pulled out by PCA. With that, we decided to visualize these two cell groups based on the relative expression of these 30 genes (by correlating the percentages of the top and bottom 15 genes in each single cell with the color channel intensities of an RGB composite image). Therefore, for every cell, its closeness to a particular group rather than an arbitrary binary classification was presented so that the transitional status of different tumor clones may be preserved. The gene list used for supervised clustering was selected based on the bulk RNA-seq expression data, in which a set of 15, out of 87, marker genes for each clone (Clone_ZNF24: *RNF146*, *DDX24*, *OAZ2*, *ZNF24*, *TXNL1*, *IDH2*, *SEPT4*, *CDCA7*, *CP*, *RAD21*, *WDR61*, *RBP1*, *COX5A*, *HSPE1*, *IER3IP1*; Clone_XIST: *XIST*, *CD44*, *FBXO32*, *LGALS1*, *ARC*, *HLA-A*, *HLA-C*, *S100A11*, *CTSV*, *SLC25A6*, *ANXA1*, *ARHGDIB*, *SQLE*, *B2M*, *NDUFS5*) in the SA501 PDX model was applied for the initial dimension reduction (to match the number of marker genes selected in unsupervised clustering). Afterwards, the major cell clusters were presented via uniform manifold approximation and projection (UMAP). To cross-check the agreement between unsupervised and supervised cell classification results, we randomly sampled 2,000 cells from the two tumor clones as annotated by either cell classification method, and determined if they were correctly assigned to the same clone by the other cell classification method (in unsupervised cell classification, a minimum level of 30% for the summed 15 marker gene counts to total gene counts was applied as the threshold for robust clone assignment).

### Imaging and image analysis

All imaging experiments were performed on a spinning disk confocal microscope (Andor Dragonfly) equipped with a Zyla sCMOS 4.2 plus camera (pixel size 6.5 μm) or a CSU-W1 SoRa super-resolution spinning disk confocal microscope (Nikon). Six main lasers on Dragonfly were used: 405 nm (100 mW), 488 nm (150 mW), 561 nm (150 mW), 594 nm (100 mW), 637 nm (140 mW) and 685 nm (40 mW). For tiled scan of full-size brain slices (Fig 1B, i and S8 Fig in S1 File), a 10× objective lens was used. For other imaging experiments, a Nikon CFI Apochromat LWD Lambda S 40XC water immersion objective lens (working distance 0.6 mm, NA 1.15) was used together with the Zeiss Immersol medium (Refractive Index 1.3339). For ExSeq using Illumina Miseq reagents, the following bandpass filters were used: 705–845 nm for base “C” channel, 663–737 nm for base “A” channel, 575–590 nm for base “T” channel, 500–550 nm for base “G” channel. All channels used 200 ms as the exposure time except that base “G” used 400 ms exposure time. For the sequenced cancer tissue, a total of 12 × 6 fields of view (FOV dimension in pre-expansion units: 104 × 104 × 62.5 μm^3^) were captured.

For characterization of expansion in uniExM, HeLa cells stained with β-tubulin antibody and DAPI were used. The size of cells was represented by the largest diameter value from the microtubule staining image, and this measurement was performed on the same cells pre- and post-expansion. In parallel, the area and shape descriptors of cell nuclei were measured with ImageJ. The following four parameters were obtained:

$$\begin{array}{c} Circularity=\frac{\left[Area\right]}{{\left[Perimeter\right]}^{2}},\;Aspect\;ratio=\frac{\left[Major\;axis\right]}{\left[Minor\;axis\right]},\\ Roundness=4\times \frac{\left[Area\right]}{{\pi \;\times \;[Major\;axis]}^{2}},\;Solidity=\frac{\left[Area\right]}{\left[Convex\;area\right]}. \end{array}$$

Quantification of expansion errors was performed as previously described [2, 3]. In brief, HeLa cells were stained with β-tubulin antibody pre-expansion. The same cells were imaged both pre- and post-expansion, where the pre-expansion images were taken with a Nikon SoRa super-resolution microscope (~1.8-time spatial resolution improvement over standard confocal microscopy). The obtained images were first histogram normalized and deconvolved in imageJ. Then non-rigid registration was performed using B-spline grids to capture potential non-uniformities between images.

For periodicity analysis of βII-spectrin, cultured neurons were stained pre-expansion. Then the cells were expanded and imaged. Segments of axon processes with more than 10 spectrin signal clusters (a signal cluster was defined as fluorescence intensity above 5 times of the standard deviation of the background level) were selected and relevant fluorescence profiles were extracted. The fluorescence traces in space were scaled back to the pre-expansion level and autocorrelation was performed in OriginLab software. From the obtained autocorrelation curve, periodicity was calculated by averaging the distances of the first four adjacent peaks.

## Results and discussion

### Epoxides as multifunctional anchors

We reasoned that an ideal multifunctional anchor for ExM should be chemically active, mechanistically predictable, and universally accessible. Epoxides fit this bill, and are already ubiquitous in daily life (*e*.*g*., in epoxy adhesives) and well understood. One study has explored polyepoxides as an intramolecular crosslinker to reduce molecular damage during harsh tissue treatments [12]. In our study, we use epoxy acrylates to directly link proteins and nucleic acids to a swellable hydrogel network permeating a specimen. The ring-opening process of epoxides is a nucleophilic substitution reaction and could follow two pathways: an S_N_1-like reaction under acidic conditions or an S_N_2 reaction under basic conditions, making the anchoring reaction pH sensitive [35]. Acidic solutions are able to protonate epoxides and open the high-tension three-atom ring directly, resulting in rapid conjugation with weak nucleophiles such as water and alcohol [36]. However, acids could also protonate nucleophiles on the biomolecules or labels to be anchored. Hence, we chose a slightly basic system, well within the range of standard specimen treatments (pH = 8.5, buffered by 100 mM sodium bicarbonate NaHCO_3_), for epoxides to react with nucleophiles of biological importance, including but not limited to cysteine, histidine, lysine, glutamic acid, tyrosine, and guanine (schematics in S2 Fig in S1 File) [12, 37, 38]. We chose for this paper to investigate glycidyl methacrylate (GMA), which contains an epoxide and a vinyl group, as an anchoring moiety. A high-level comparison of LabelX, MelphaX, and GMA with regards to key properties in application is summarized in S1 Table in S1 File.

We confirmed that uniExM is compatible with both a popular protein retention form of ExM (proExM) and an RNA retention form of ExM that supports *in situ* hybridization (ExFISH) (examples in S3 Fig in S1 File and Fig 1B, i). Specifically, 0.04% (w/v) GMA, as compared to an established method using 0.01% (w/v) LabelX plus an additional 0.005% (w/v) AcX [4], fared well, retaining native yellow fluorescent protein (YFP) intensity ~4 times stronger (Fig 1B, ii). The single-spot intensity of hybridization chain reaction (HCR)-FISH was 2 times stronger in GMA treated tissues (Fig 1B, ii), reminiscent of the improvement yielded by MelphaX over LabelX [27]. Both RNAs and proteins could be retained and visualized in the same specimen, after GMA anchoring, for both cultured cells and intact tissues (Fig 1B, iii); hence, we termed this simplified protocol united expansion microscopy (uniExM).

### Characterization of uniExM distortion and yield

Perhaps because only the anchoring step was changed, without alteration of the gelation, softening, and expansion steps, the rest of the expansion process proceeded smoothly, without requiring further refinement. Not surprisingly, uniExM supported high-resolution imaging of fine structures, including microtubules in cultured HeLa cells (Fig 2A). The expansion factor was found to be 4.2–4.4 using 0.04% GMA (Fig 2A, iv) and the distortion incurred by expansion was similar to previously published results, a few percent over a field of view of a few tens of microns (benchmarked against Nikon SoRa super-resolution microscopy) (Fig 2A, v). In addition, the morphology and geometry of the nucleus was reliably maintained after expansion (S4 Fig in S1 File).

As with HCR-FISH before it, uniExM is able to decrowd densely packed RNA molecules for better detection (Fig 2B, i). To gauge how uniExM facilitates retention of RNA molecules, we systematically varied GMA concentration, reaction pH, and temperature, evaluating the retention of three highly expressed genes (*GAPDH*, *EEF1A1*, *ACTB*) (S5 Fig in S1 File). The optimal reaction condition was determined to be 0.04% GMA for cultured cells at pH 8.5 (100 mM NaHCO_3_), at room temperature. For tissue samples we used 0.1% GMA to ensure sufficient anchoring of multiple types of biomolecules, and found in practice that increased temperature (*e*.*g*., from room temperature to 37°C) could facilitate the diffusion and anchoring efficiency of epoxide, consistent with previous reports [4, 6]. Moreover, we demonstrated that the anchoring reaction could be efficiently controlled by varying temperature and pH; at 4°C and neutral pH, the anchoring efficiency for RNA can be suppressed by more than 50% after 12 h incubation (termed reaction “Off” condition), while it can be rapidly recovered to full efficacy by an additional 3 h incubation at 25°C and pH 8.5 (termed reaction “On” condition) (S5D Fig in S1 File).

We quantified three moderately expressed genes (*TOP2A*, *TFRC*, *USF2*) with HCR-FISH before vs. after GMA anchoring. uniExM preserved RNA molecules with ~100% retention efficiency (Fig 2B, ii, iii). As another comparison, GMA was equal to, or perhaps even slightly better, than LabelX in preservation of highly expressed mRNA targets (S6 Fig in S1 File). Thus, both in terms of supporting even expansion, and high yield of target, GMA is an excellent unified anchor for both proteins and RNAs.

### uniExM for preservation of protein content and ultrastructure

We next explored applications of uniExM in different biological contexts. We chose βII-spectrin in neurons to image, since its periodic distribution in axons was discovered via super-resolution imaging [39, 40]. In cultured mouse hippocampal neurons, the periodic distribution of βII-spectrin was prevalently observed in axons (Fig 3A; more examples in S7A Fig in S1 File). The distance between two adjacent βII-spectrin spots was found to be ~190 nm (Fig 3B), as reported earlier [39], while this periodicity was not apparent in pre-expansion samples. The periodic distribution of βII-spectrin can be additionally visualized with an autocorrelation analysis (Fig 3C), which we performed with a 7× expansion protocol based on the TREx protocol [26]. Different expansion factors can be obtained by tuning the cross-linker concentration of the expansion microscopy hydrogel; here we simply show the compatibility of our new anchoring strategy with the tuning of hydrogel expansion factor. Finally, autocorrelation analyses from four independent samples were performed, yielding calculated periodicity values (193 ± 15 nm for 4× expanded cells, 187 ± 10 nm for 7× expanded cells, values reported as mean ± standard deviation) consistent with results previously obtained by super-resolution STORM or STED imaging (Fig 3D and S7B Fig in S1 File) [41, 42]. Moreover, post-expansion antibody staining revealed the same spatial pattern of βII-spectrin in both 4× and 7× expanded cells (Fig 3E and S7C Fig in S1 File). Post-expansion staining is increasingly popular because expanding proteins away from each other, and then staining them, can make more room for antibodies to bind to their targets [25]; here, we simply show compatibility of our new anchoring strategy with post-expansion staining.

Polyepoxides, which can crosslink multiple biomolecules to each other, and even multiple parts of a biomolecule to each other, have been shown to help proteins such as fluorescent proteins retain function in tissue specimens under harsh conditions, such as high heating [12]. High heat treatment of hydrogel-embedded tissues is a popular softening method to precede an expansion step, because it preserves epitopes for post-expansion staining, which can result in better access to epitopes than pre-expansion staining [25, 43]. However, the high heat treatment can destroy the fluorescence of GFP, amongst other sensitive moieties. We combined GMA with a polyepoxide, trimethylolpropane triglycidyl ether (TMPTE), to assess the performance of this cocktail in preservation of fluorescent protein function after heat-based tissue softening. We tested two tissue processing protocols requiring heating above 50°C: the SDS-based sample denaturation (95°C for 1h) [25] and the proK-based enzymatic digestion (60°C for 2h) [44]. Compared with LabelX plus AcX, GMA plus TMPTE showed better retention of fluorescence signals for paraformaldehyde (PFA)-perfused, fresh frozen Thy1-YFP mouse brain slices (~440% signal improvement for SDS-based denaturation; 250% signal improvement for proK-based digestion; S8 Fig in S1 File).

### uniExM for *in situ* sequencing of RNA

**Fig 4:**
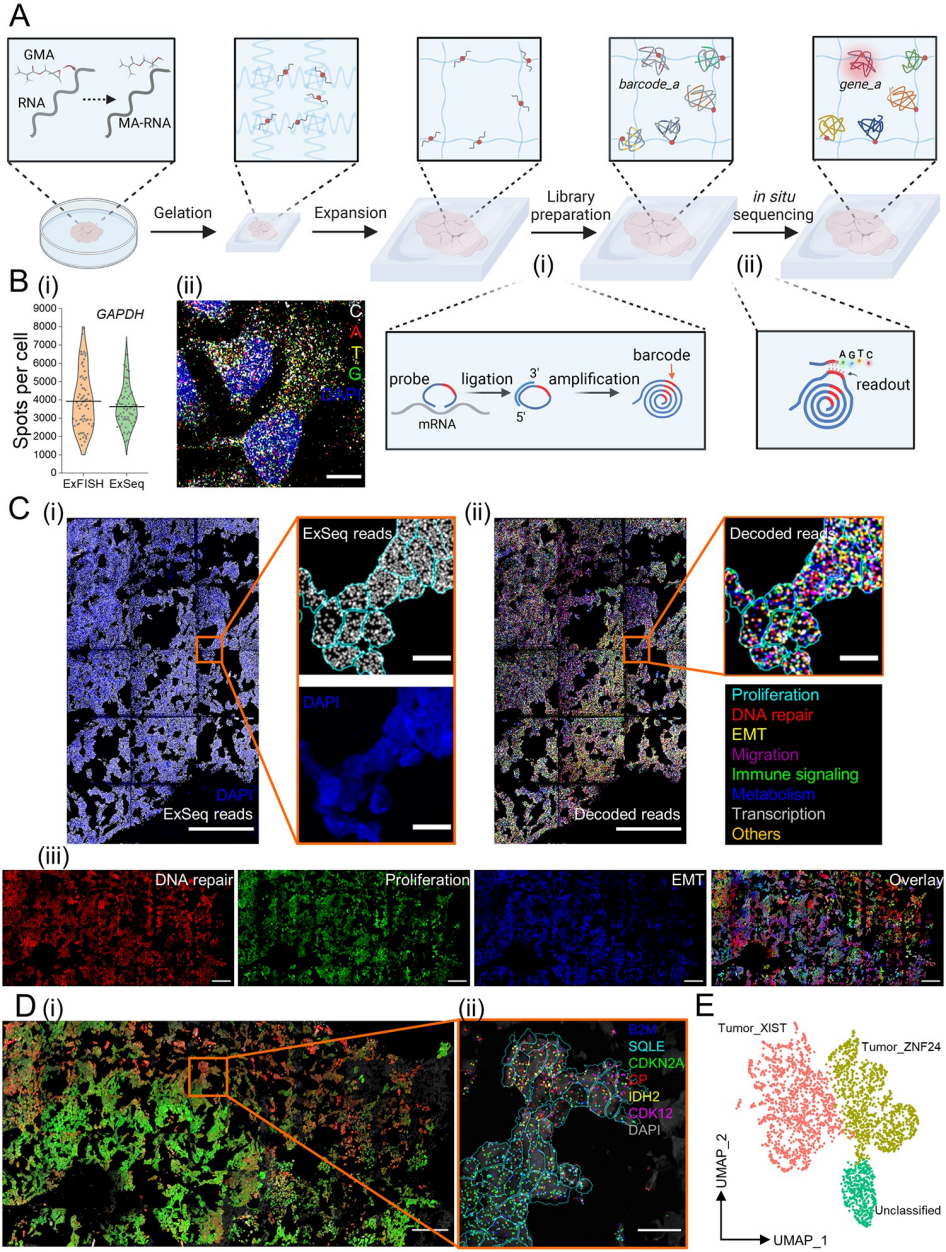
uniExM-supported *in situ* RNA sequencing (ExSeq). **(A)** Schematic of the workflow for targeted ExSeq (tExSeq): target RNA molecules are reacted with GMA to acquire methacrylate groups (termed MA-RNA) and anchored to the expandable hydrogel. Padlock probes are then introduced to hybridize with the target RNAs in the expanded biological sample. Upon successful hybridization, the sequence of a target mRNA serves as a “splint” for PBCV-1 enzyme-mediated ligation of the bound padlock, as shown in the zoomed-in panel **(i)**. Afterwards, rolling circle amplification (RCA) is applied to amplify the ligated probes that harbor barcodes specific to targets. Finally, the barcodes are read out by *in situ* sequencing chemistry, as shown in the zoomed-in panel **(ii)**. The full readout of a specific barcode can then be used to reveal the gene identity (*e*.*g*., from barcode_a to gene_a) together with its location information. In such way, multiple gene targets can be decoded (represented as differentially colored amplicons). **(B)** Validation of ExSeq enzymatics and sequencing-by-synthesis (SBS) chemistry in samples processed with the uniExM procedure. **(i)** *GAPDH* in HeLa cells was chosen to undergo HCR-FISH or tExSeq. The numbers of detected signal spots per cell were quantitatively compared. No statistically significant difference was observed between the two methods. (Data shown as violin plots, with raw data points presented, and mean values highlighted with solid lines; n = 70 cells from 4 samples, 2 culture batches; two-sample *t*-test was performed with *p >* 0.1) **(ii)** A fluorescence image showing raw ExSeq signals from all four base channels in HeLa cells undergoing targeted ExSeq. Scale bars (in pre-expansion units): 20 μm. **(C)** Demonstration of ExSeq applying an 87-gene panel in GMA-anchored SA501 PDX breast cancer tissue. **(i)** Overview of the raw ExSeq reads (gray spots) in the tissue. DAPI staining for nuclei was used for cell segmentation and reads assignment (shown in the zoomed-in images). **(ii)** The raw ExSeq reads were decoded and colored based on 8 distinct gene function groups (full list in S5 Table in S1 File). Scale bars (in pre-expansion units): 100 μm (for stitched overview images), 10 μm (for zoomed-in images). **(iii)** Gene maps of 3 selected function groups—DNA repair, proliferation and epithelial-mesenchymal transition (EMT), were used to help visualize heterogeneity of cell status in the whole tissue. The decoded transcripts of genes belonging to each functional group were summed and their ratio to total transcript counts were assigned to the “R”, “G”, “B” color channels of the image, respectively. During the color assigning process, a scaling factor of 3.33 (for DNA repair and proliferation) or 2.5 (for EMT) was introduced; that is, if the EMT group of genes was 40% of the total transcripts in one cell, its assigned “B” channel was given the maximum color intensity (40% X 2.5 = 100%). Then, the three individual channels were combined to make a composite image (right). Scale bars (in pre-expansion units): 100 μm. Linear expansion factor: 3.2 (the expanded gel was re-embedded before sequencing). **(D)** Unsupervised principal component analysis (PCA) identified two primary gene groups for cell classification. **(i)** Using these two PCA gene groups, a distribution of different cancer cells was revealed. In this presented image, the summed transcripts of each PCA group normalized to the total transcript count for a given cell were assigned to the “R” channel and “G” channel, respectively (scaling factor: 3.33). Then the two images were overlaid to make a composite. Scale bar (in pre-expansion units): 100 μm. **(ii)** In the zoomed-in region where differentially colored cells co-exist, 6 marker genes are plotted; their distribution varies across cells in the subregion. Scale bar (in pre-expansion units): 10 μm. **(E)** Uniform manifold approximation and projection (UMAP) representation of the cell typing results using bulk RNA-seq identified marker genes in the SA501 cancer model. According to the panel design, the 87 gene list could differentiate two primary cancer cell clones that are successfully annotated on UMAP—“Tumor_XIST” and “Tumor_ZNF24”, named after their feature genes. A small group of cells are marked as “Unclassified”, likely attributed to non-cancer interstitial or other cells.

Expansion microscopy greatly facilitates *in situ* sequencing, enabling multiplexed RNA analysis with high spatial precision; we named the optimized combination expansion sequencing (ExSeq) [21]. In untargeted ExSeq, linear probes containing randomized octamer sequences are hybridized to RNA targets, followed by reverse transcription of adjacent RNA sequence information into cDNA form. cDNAs are then circularized and undergo rolling circle amplification (RCA) (schematized in S9 Fig in S1 File). Then, in each round of sequencing readout, the bases being added are imaged through fluorescence microscopy (example in S10A Fig in S1 File). In addition to untargeted ExSeq, one can perform targeted ExSeq against sets of specific RNAs (Fig 4A), by bringing in padlock probes that hybridize to targets, and then sequencing barcodes found on the padlock probes (schematized in S9 Fig in S1 File; example in S10B Fig in S1 File). Comparing amplicons (containing amplified barcode sequences) from targeted ExSeq using GMA, to spots seen with classical HCR-FISH, showed excellent agreement in spot counts (Fig 4B, i). The sequencing, conveniently, can be done with standard Illumina MiSeq sequencing-by-synthesis (SBS) reagents (Fig 4B, ii). We performed sequencing of barcodes on padlock probes targeting *ACTB* mRNAs to test the stability of uniExM-based ExSeq across multiple rounds of sequencing in mouse brain tissues. The padlock probes used contained repetitive “T” bases in their barcode region, so the amplicons should emit the same fluorescence signal in each round of sequencing. The amplicons were first examined with a universal detection probe to generate a reference image, then three consecutive rounds of sequencing were performed (S10C Fig in S1 File). Amplicons were consistently detected in each round (with an average detection rate of ~96.5% across 20 fields of view from 2 different mouse brains), indicating that uniExM-based ExSeq is compatible with the full SBS chemistry cycle (*i*.*e*., elongation, detection and cleavage steps). Moreover, Thy1-YFP positive structures in the mouse brain tissues (*e*.*g*., dendrites and spines) were consistently detected after the ExSeq library preparation procedure (as seen by comparing images from the YFP channel before vs. after library preparation), helpful for analyzing transcriptomics in the context of morphological compartments like synapses and axons.

We performed 7-round SBS to profile 87 genes known to classify tumor cells into subclasses in SA501 breast cancer PDX tissues [30] (the original LabelX-based ExSeq was demonstrated for 4 rounds of SBS). Importantly, uniExM significantly reduces the cost of ExSeq, as the anchoring reagent contributes to more than half of the entire cost of the original protocol (see S1 Table in S1 File for more details). In a 1.24 × 0.62 mm^2^ region of interest, 793,535 raw reads (colored by function annotations) and 3,339 cells in total were detected (Fig 4C, ii and S10D Fig in S1 File). Many kinds of analysis are possible: in Fig 4C, iii, transcripts from three functional groups were presented as 8-bit “RGB” images where the color intensity reflected the overall expression level of the members of that group in each individual cell. Principal component analysis (PCA), applied to all the cells of the sample, revealed two distinct cell groups distinguished by 30 out of the 87 genes (S10E Fig in S1 File). Based on the expression levels of these two PCA-generated gene sets, we assigned a color code to each individual cell (Fig 4D, i). The summed counts of each PCA group-specific gene set (15 genes), normalized to the total count in each individual cell, were then plotted as the red and green channels of the “RGB” image, respectively. The composite image shows a spatially varying distribution of the colored cells in space, explicitly delineating two tumor cell populations. In a zoomed-in region where cells of both groups were present, transcripts from distinct PCA lists were detected in different cells (Fig 4D, ii). As a second method of analysis, we ran a dimensionality reduction algorithm using the clone-specific marker genes with significant differential expression from the RNA-seq data of the same PDX model [34] and presented the results with UMAP, where two tumor cell clones (denoted as “Tumor_XIST” and “Tumor_ZNF24”) and one group of “Unclassified” cells were identified (Fig 4E). These two classified groups of cells well correspond to the bright-green and bright-red colored cells in Fig 4D, i, respectively. In detail, comparing the unsupervised and supervised results, 85.1% of tumor clone cells annotated by the supervised method were classified as the same clone by unsupervised method, whereas 82.7% of cells classified by the unsupervised method were cross-verified by the supervised method. In this demonstration, starting from a marker list selected either by unsupervised PCA or supervised RNA-seq data, highly consistent tumor clone identification was obtained. These orthogonal analyses not only help support the validity of the biological findings but also reflect the robustness of our technology.

### uniExM for multimodal detection beyond proteins and nucleic acids

Our focus here was the retention of proteins and RNAs with a single anchor during ExM. Without seeking to make broad quantitative claims about the efficacy of doing so, we did examine at a qualitative level the compatibility of uniExM with commercially available lipid stains and markers of carbohydrates. We selected three lipid stains—octadecyl rhodamine B chloride (R18), FM 1-43FX (FM) and BODIPY FL C_12_ (BODIPY), all of which showed signals in lipid-rich or membranous structures (S11A, S11B Fig in S1 File). consistent with their staining patterns in unexpanded samples (S12A Fig in S1 File). In addition, GMA treated cells showed significantly stronger lipid staining signals than AcX treated cells (S12B Fig in S1 File). Using Alexa647 tagged wheat germ agglutinin (WGA), which binds *N*-acetylglucosamine (GlcNAc), we were able to see specific signals in nuclear membranes of cultured HeLa cells and blood vessels of mouse brain tissues (S11C and S13 Figs in S1 File). Notably, as WGA can specifically bind to glycoprotein-enriched nucleoporins (a key component of the nuclear pore complex) [45, 46], GMA efficiently detected putative nucleoporin puncta on nuclear membranes in the context of uniExM (S12C Fig in S1 File). Such stains could potentially be used together, in the same specimen, along with antibody stains and FISH probes, supporting multimodal imaging of many kinds of biomolecule in the same cell or tissue section (S13 Fig in S1 File).

## Conclusions

Here we show that a single anchoring molecule can support the expansion of both proteins and RNAs away from each other in expansion microscopy, reducing the complexity and cost compared to earlier anchoring strategies, which required different anchors for different kinds of biomolecules. We used an epoxide, an inexpensive and highly reactive moiety capable of binding to many kinds of biomolecules, that contained a vinyl group capable of participating in polymerization. We found this molecule, GMA, to enable good retention of proteins and RNAs, and to support labeling for visualization of other biomolecules, although more work will be required to quantitatively validate the latter in multiple cell and tissue types.

Past anchoring strategies for ExM have used different anchors for different kinds of biomolecules, *e*.*g*., using an aldehyde or NHS ester to bind amines on proteins, or an alkylating reagent to bind guanine on RNA. The latter strategy requires end users to mix multiple chemicals overnight to create the anchor, and some protocols administer different anchors in separate steps. Here, no end user synthesis is needed, and only a single step is needed to add the multimodal anchor GMA.

We demonstrated the utility of this united ExM, or uniExM, protocol in cells and tissues. Perhaps because of the rest of the protocol is unaltered compared to previous ExM protocols, we saw no differences in the performance of the rest of the protocol (*e*.*g*., distortion, resolution). uniExM was able to support not just classical ExM protocols like antibody detection of proteins and FISH detection of RNAs, but also our recently published ExSeq protocol for multiplexed visualization of RNAs. In short, uniExM will find utility in a variety of contexts in biology.

## Supporting information

S1 File Document containing S1–S13 Figs and S1–S5 Tables. (DOCX)
