# Supplementary material for: Expansion microscopy using a single anchor molecule for high-yield multiplexed imaging of proteins and RNAs
Source: PLoS One. 2023 Sep 20;18(9):e0291506. doi: 10.1371/journal.pone.0291506 (PMC10511132; doi:10.1371/journal.pone.0291506)
Supplement: S1 File — (DOCX) [file pone.0291506.s001.docx]

**SUPPORTING INFORMATION**

**Expansion Microscopy Using a Single Anchor Molecule for High-Yield Multiplexed Imaging of Proteins and RNAs**

Yi Cui^1,2¶,#a^, Gaojie Yang^1,2¶^, Daniel R. Goodwin^1,2^, Ciara H. O'Flanagan^3^, Anubhav Sinha^1,2,4^, Chi Zhang^1,2^, Kristina E. Kitko^1,2,#b^, Tay Won Shin^1,2^, Demian Park^1,2^, Samuel Aparicio^3,5^, CRUK IMAXT Grand Challenge Consortium^^^, Edward S. Boyden^1,2,7,8,9,10*^

^1^McGovern Institute, Massachusetts Institute of Technology (MIT), Cambridge, MA, USA

^2^Media Arts & Sciences, MIT, Cambridge, MA, USA

^3^Department of Molecular Oncology, BC Cancer, Vancouver, BC, Canada

^4^Harvard-MIT Program in Health Sciences and Technology, MIT, Cambridge, MA, USA

^5^Department of Pathology and Laboratory Medicine, University of British Columbia, Vancouver, BC, Canada

^7^Department of Biological Engineering, MIT, Cambridge, MA, USA

^8^Department of Brain and Cognitive Sciences, MIT, Cambridge, MA, USA

^9^Koch Institute for Cancer Research, MIT, Cambridge, MA, USA

^10^Howard Hughes Medical Institute, MIT, Cambridge, MA, USA

^*^Corresponding author: [edboyden@mit.edu](mailto:edboyden@mit.edu)

^¶^These authors contributed equally to this work

^#a^Current address: NanoString Technologies, Inc., Seattle, WA, USA

^#b^Current address: Eli Lilly and Company, San Francisco, CA, USA

^^^List of the IMAXT Consortium investigators is provided in the Acknowledgements.

**Supplementary Figures**


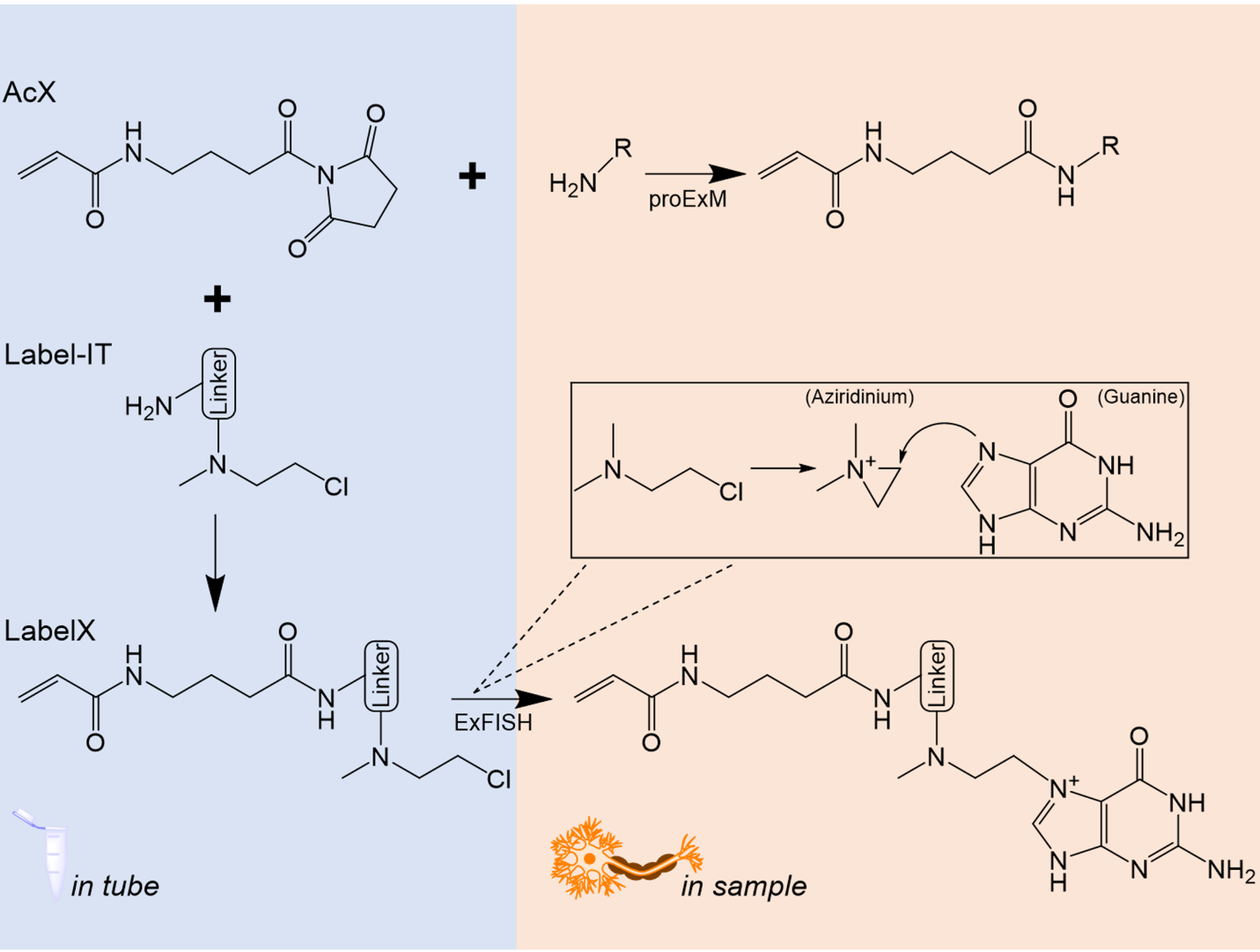


**S1 Fig. Working mechanisms of the anchoring chemistry of standard ExM.** In protein-retention ExM (proExM), AcX functions by modifying the amine groups on proteins through a succinimidyl ester moiety. The acryloyl group of AcX crosslinks the proteins to the polyacrylate hydrogel. In order to anchor nucleic acids, AcX is first reacted with an alkylating reagent such as Label-IT amine to form aziridinium-containing LabelX which can later be coupled to the N7 position of guanine in DNA and RNA. However, this in-house synthesis of anchor molecules suffers from high cost, nonstandard yield, and increased procedure time and complexity.


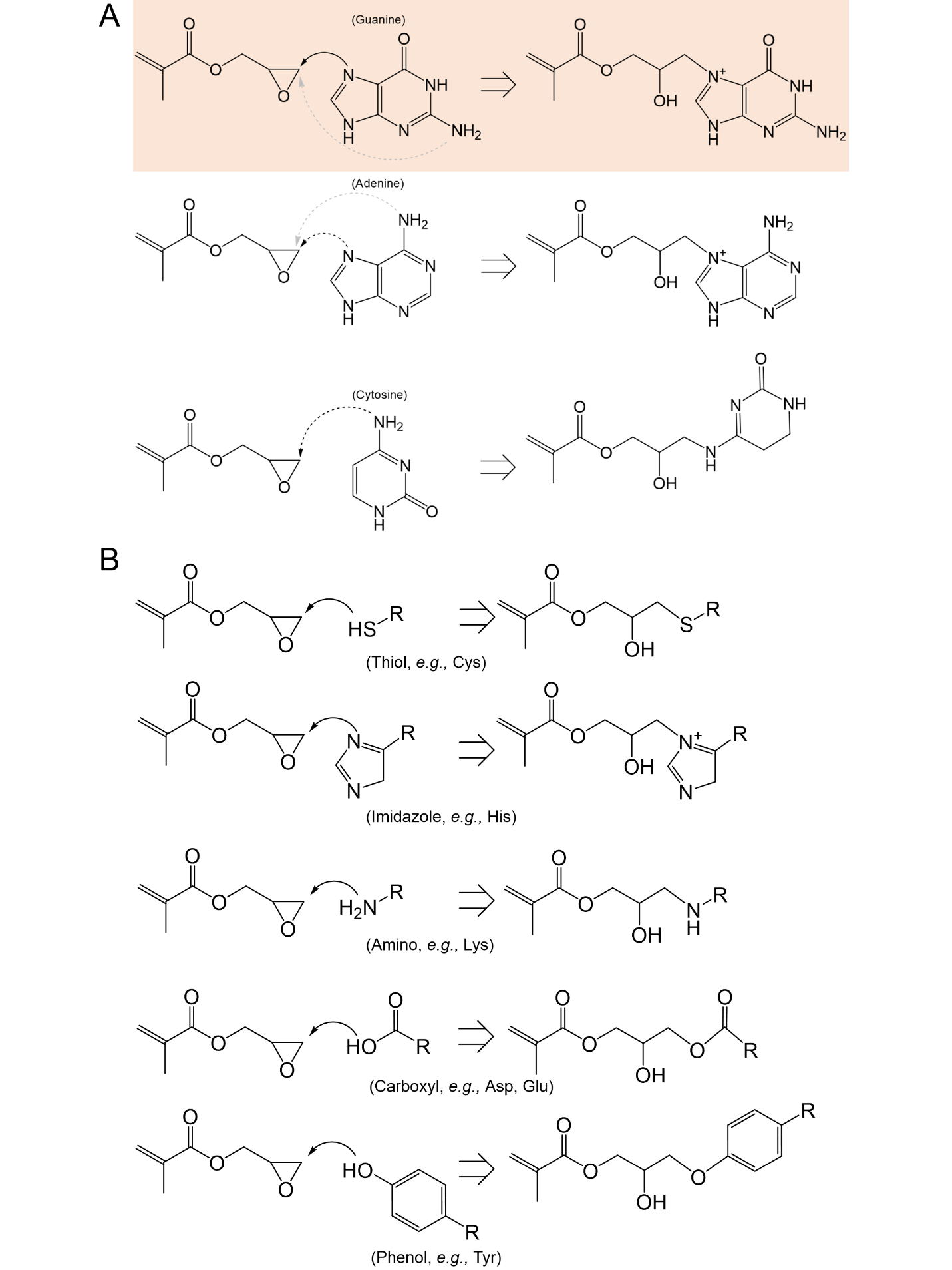


**S2 Fig. Representative potential nucleophile substrates in a biological system,** including **(A)** nucleic acids and **(B)** amino acids. A potential reaction between GMA and N7-guanine in anchoring DNA and RNA is highlighted in orange. Potential reactions between common nucleophilic amino acids and GMA are illustrated.


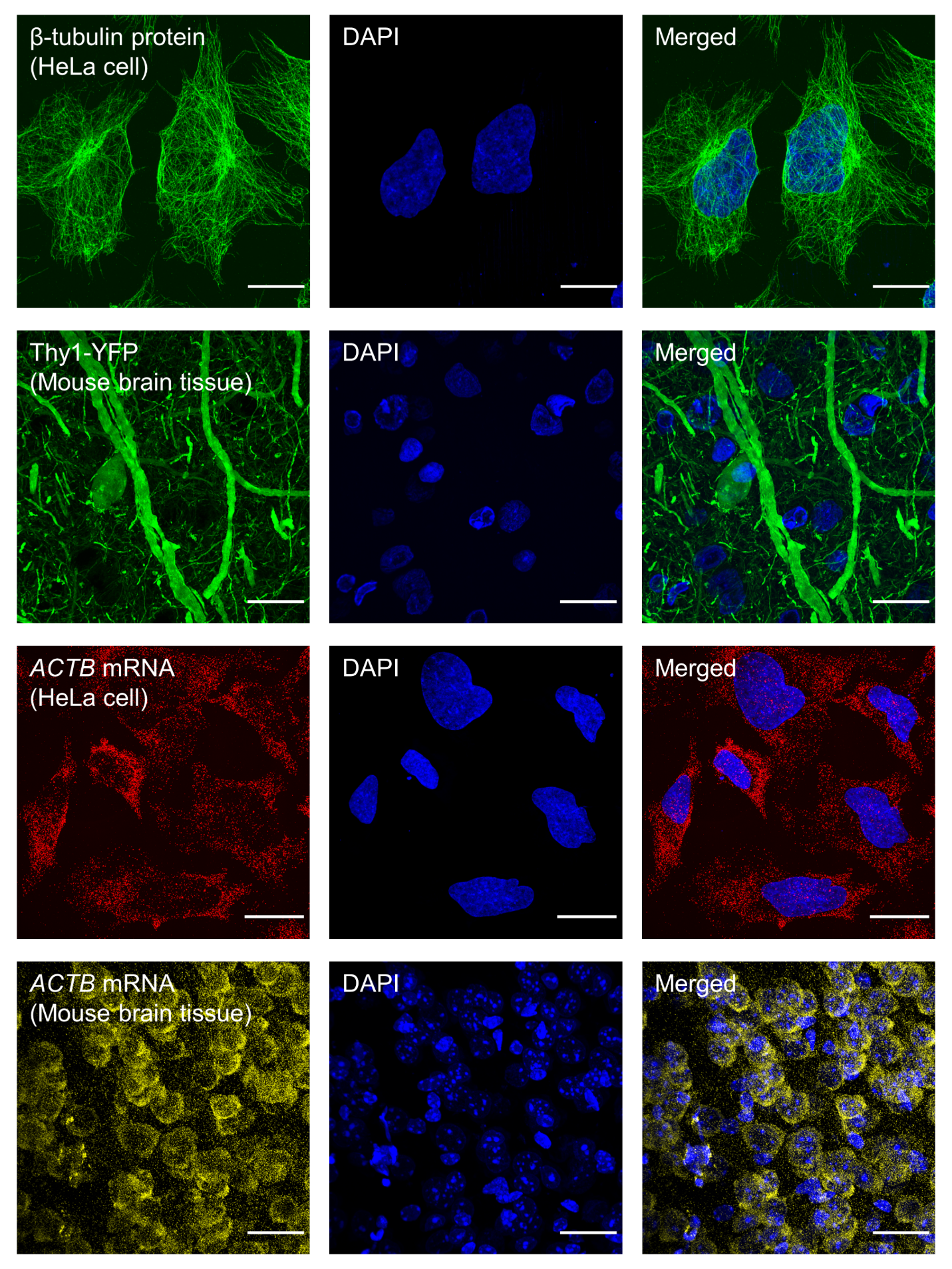


**S3 Fig. GMA-based uniExM enables retention of proteins and RNAs in expanded cells and tissues.** In protein retention tests, β-tubulin in HeLa cells and YFP in mouse brain tissues were chosen as targets. Row 1: HeLa cells were anchored with 0.04% (w/v) GMA, digested with LysC and stained with anti-β-tubulin (and DAPI) post-expansion. Row 2: A 50 µm thick Thy1-YFP mouse brain slice was anchored with 0.1% (w/v) GMA, digested with proK, and imaged post-expansion. In RNA retention tests, HCR-FISH targeting *ACTB* in HeLa cells (Row 3) and mouse brain tissues (Row 4) were performed post-expansion, respectively. Color representation in the images: blue – DAPI; green – Thy1-YFP/Alexa488; yellow – Alexa546; red – Alexa647. Scale bars (in pre-expansion units): 20 µm.


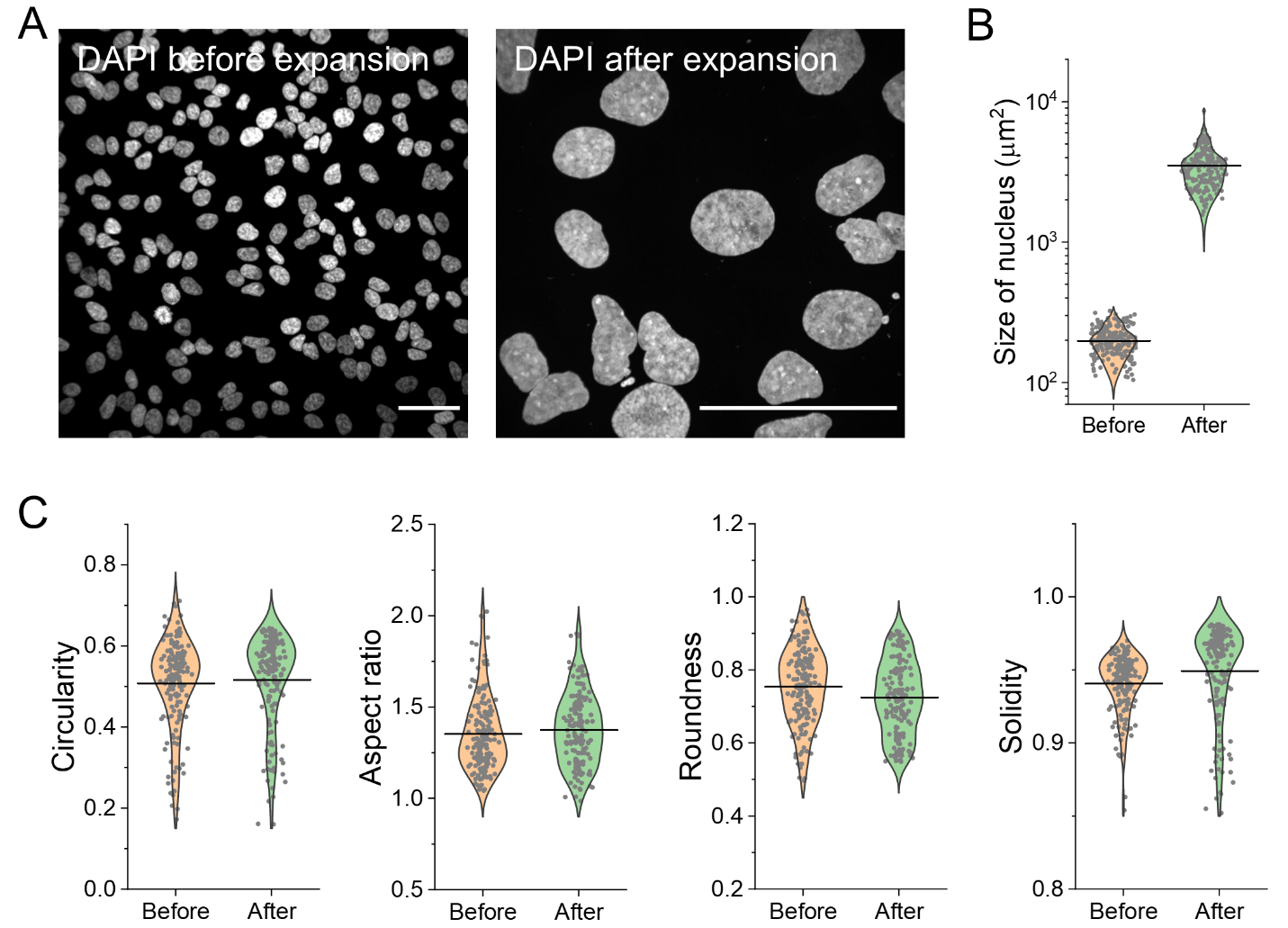


**S4 Fig.** **Assessment of the size and morphological properties of HeLa cell nuclei before and after GMA-based uniExM. (A)** Representative images of DAPI staining for HeLa cell nuclei before and after expansion. Scale bars (in pre-expansion units): 50 µm. **(B)** Size of nuclei was measured before and after expansion. **(C)** 4 parameters related to nuclear morphological properties – circularity, aspect ratio, roundness and solidity – were evaluated within ImageJ. (Data presented in violin plots with raw data points shown and mean values highlighted with solid lines, n = 200 cells from 4 culture batches, two sample *t*-test was performed with all *p* > 0.1)


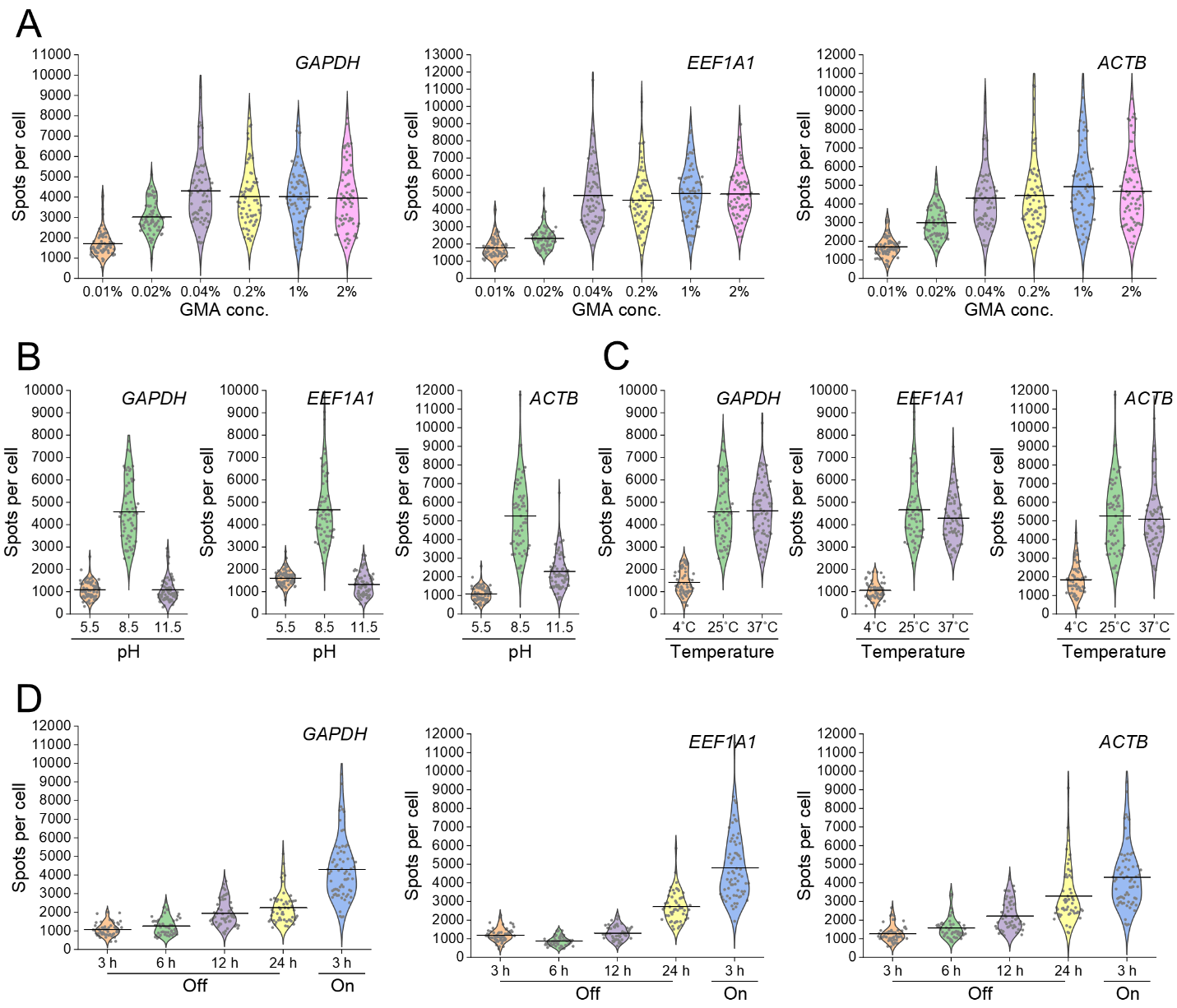


**S5 Fig.** **Optimization for GMA-based RNA anchoring in uniExM. (A)** Different concentrations of GMA, **(B)** anchoring pH and **(C)** temperatures were tested in the context of ExFISH targeting three genes – *GAPDH, EEF1A1 and ACTB* – in HeLa cells, to determine optimal RNA retention conditions. **(D)** In light of the above tests, the GMA anchoring reaction for RNAs could be tuned “On” and “Off” by varying the reaction temperature and pH. Reaction “Off” condition: 4°C, pH = 7.4 with 1×PBS. Reaction “On” condition: 25°C, pH = 8.5 with 100 mM NaHCO_3_. To test the “Off-On” transition, one group of cells were first incubated under the “Off” condition for 12 h, followed by treatment under the “On” condition for 3 h. (n = 50-80 cells for each tested condition and from 2 culture batches)


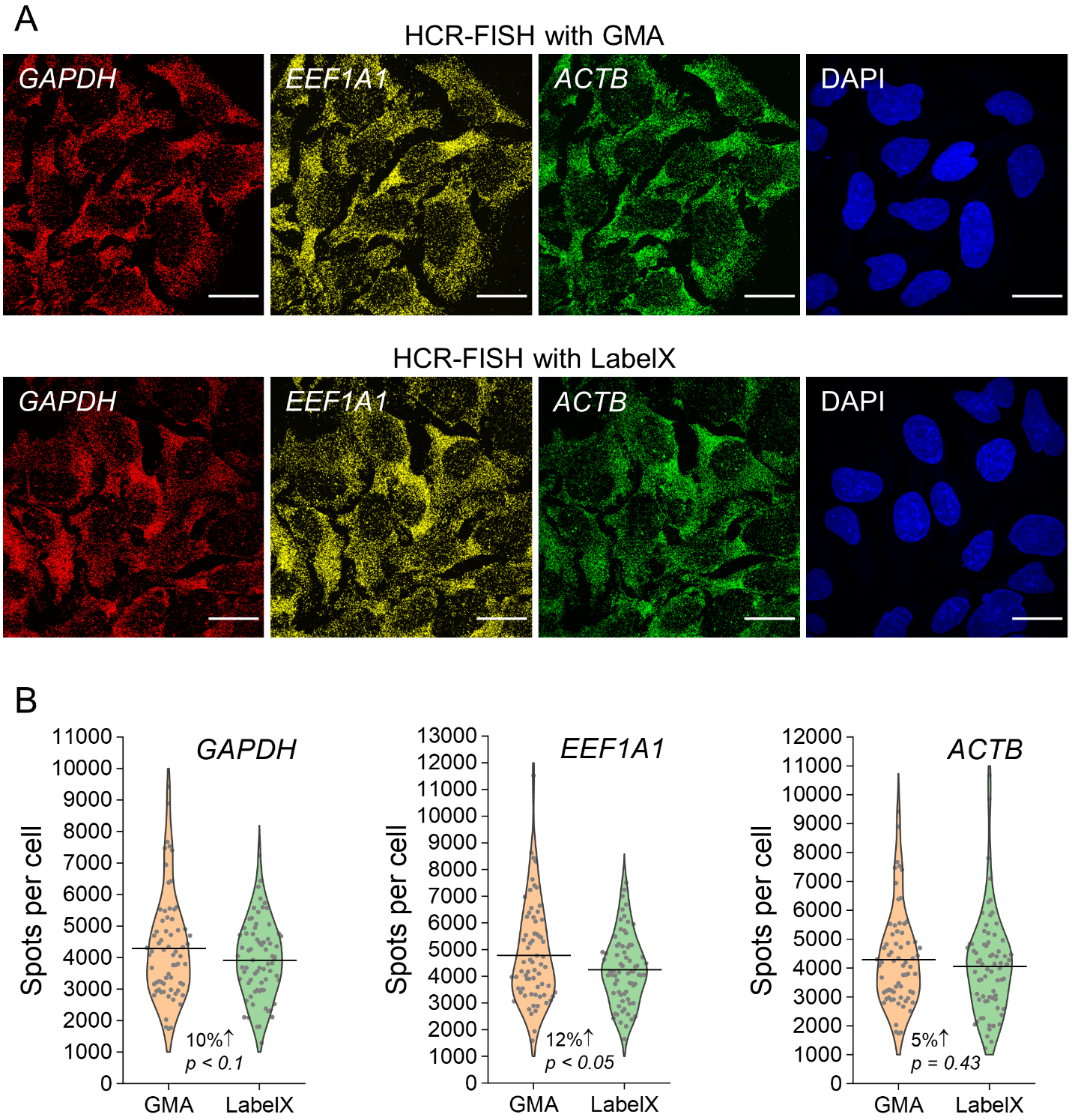


**S6 Fig.** **Head-to-head comparison between GMA and LabelX in RNA retention and detection. (A)** HCR-FISH targeting three highly expressed housekeeping genes was performed in HeLa cells treated with 0.04% (w/v) GMA or 0.01% (w/v) LabelX, under their respective optimal conditions. Color representation in the images: blue – DAPI; green – Alexa488; yellow – Alexa546; red – Alexa647. Linear expansion factor: 4.3 for GMA; 4.4 for LabelX (prior to re-embedding). Scale bars (in pre-expansion units): 20 µm. **(B)** Summary plots of detected transcripts per cell for each gene. (Data presented in violin plots with raw data points shown and mean values highlighted with solid lines; n = 70-100 cells from 3 culture batches; two-sample *t-*test was performed, with *p* values shown)


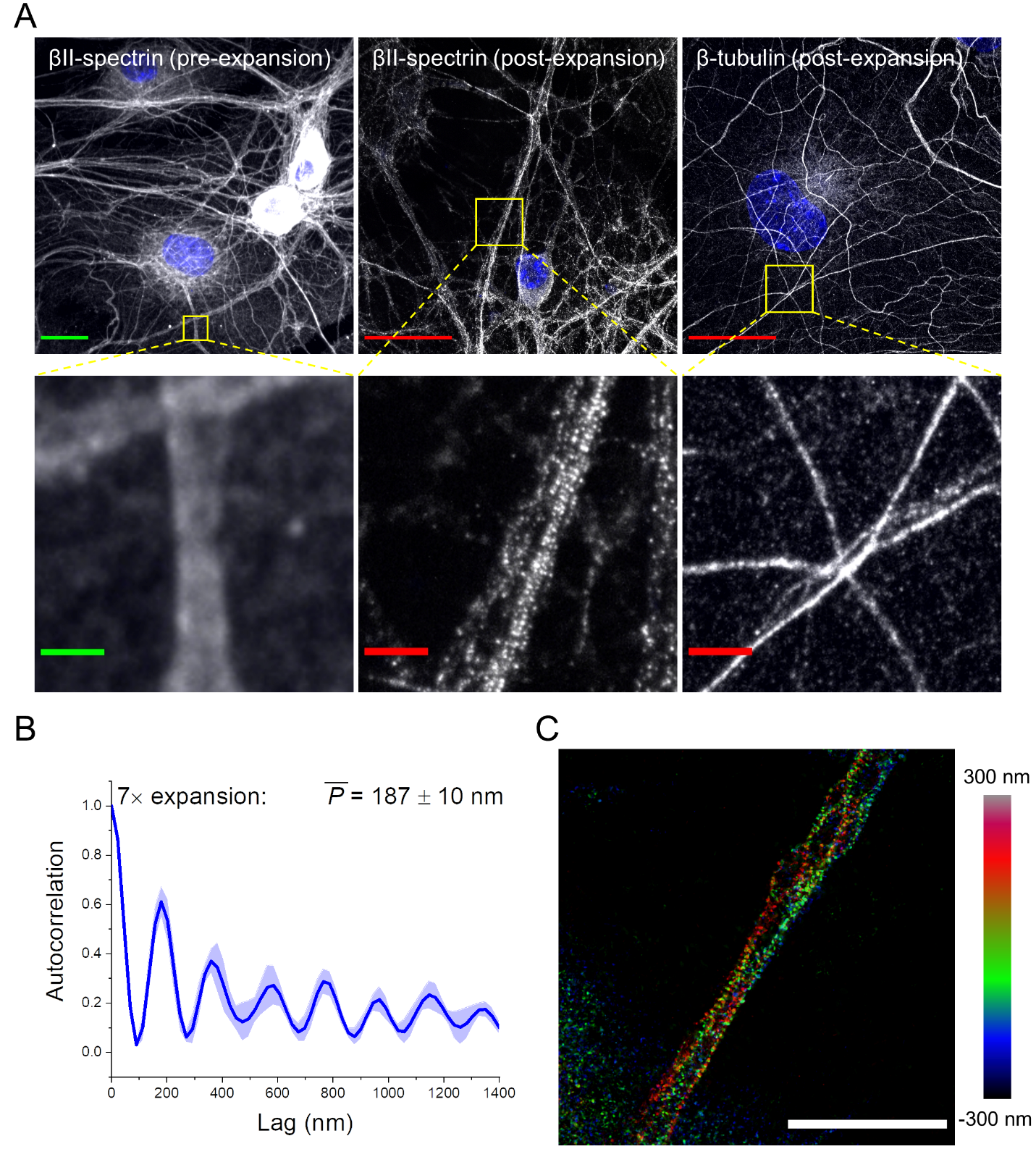


**S7 Fig.** **uniExM helps resolve the ultrastructure of βII-spectrin in neuron axons – additional data.** Left column: antibody staining for βII-spectrin in mouse hippocampal neurons shows apparently continuous signal distribution along neuronal processes. Middle column: the periodic, punctate distribution of βII-spectrin signals is revealed, after expansion. Right column: antibody staining against β-tubulin shows continuous microtubule structures even with expansion. All antibody staining was performed pre-expansion. Linear expansion factor: 4.4. Color representation in the images: gray/white – antibody staining; blue – DAPI. Scale bars (in pre-expansion units): 20 µm (upper panels); 2 µm (lower panels). **(B)** Mean autocorrelation function of periodicity analysis using 7× expansion. (Solid lines, mean; shaded areas, standard error of mean. n = 40 measurements from 2 culture batches). **(C)** Post-expansion antibody staining revealed the same periodic distribution pattern of βII-spectrin under 7× expansion. Scale bar (in pre-expansion units): 5 µm. The color code of the image represents z-axis information.


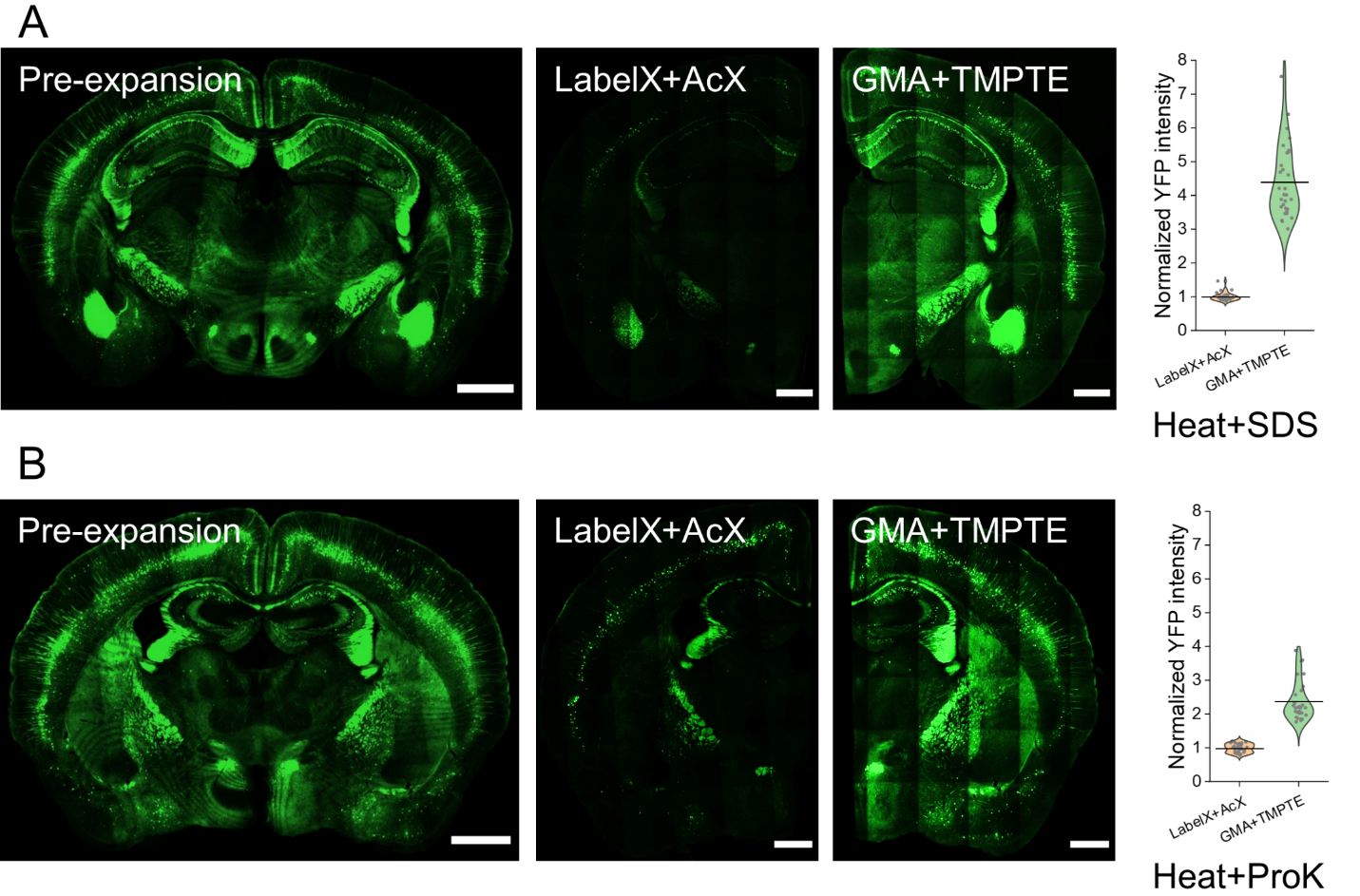


**S8 Fig.** **Preservation of fluorescent protein function in tissues undergoing high heat treatments by combining acrylate epoxides and polyepoxides.** Retention of Thy1-YFP fluorescence signals in mouse brain tissues by LabelX plus AcX vs. GMA plus TMPTE after: (A) strong detergent SDS-based denaturation: 95°C for 1 h, followed by 37°C overnight; (B) ProK-based digestion: 60°C for 2 h, followed by 37°C overnight. Scale bars (in pre-expansion units): 1,000 µm (whole brain); 500 µm (half brain). Normalized YFP intensity measurements are presented in violin plots with mean values highlighted with solid lines. The intensity values were normalized to the level of LabelX plus AcX treated samples. (n = 30 measurements from 4 brain slices, 2 mouse brains; two-sample *t*-test was performed for statistical significance tests, with both *p* < 10^-15^)


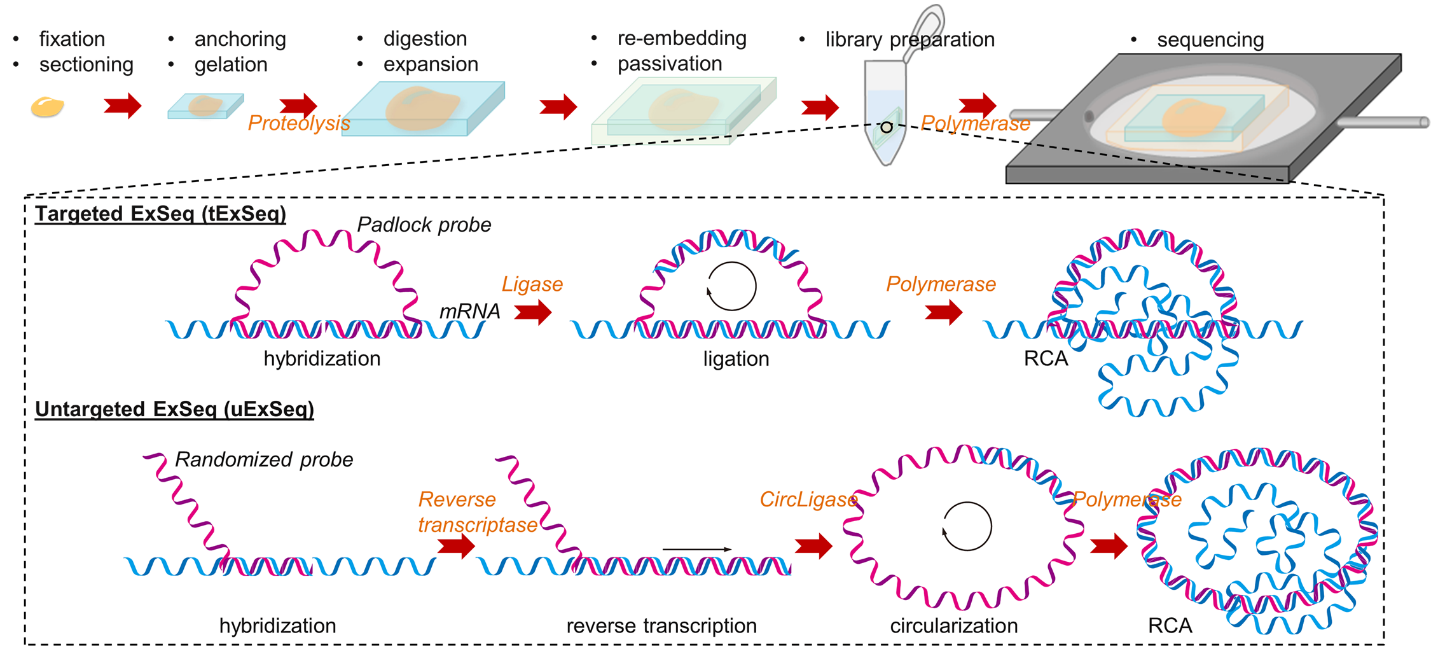


**S9 Fig.** **Schematic illustration for targeted and untargeted ExSeq procedures.** After a sample is fixed, sectioned, and expanded, it undergoes a second gelation in a charge-neutral hydrogel (the process of “re-embedding”) followed by neutralization of charge on the original hydrogel (the process of “passivation”) to prepare the sample for sequencing. Then padlock probes targeting specific mRNAs (for tExSeq) or randomized oligonucleotide probes (for uExSeq) are introduced. In tExSeq, the padlock probes are directly ligated upon hybridization to their designated targets, while in uExSeq the randomized probes prime reverse transcription to add sequence information from the bound RNA into cDNA form, followed by probe circularization. The ligated or circularized probes are then subjected to rolling circle amplification (RCA) before being sequenced by ligation or synthesis.


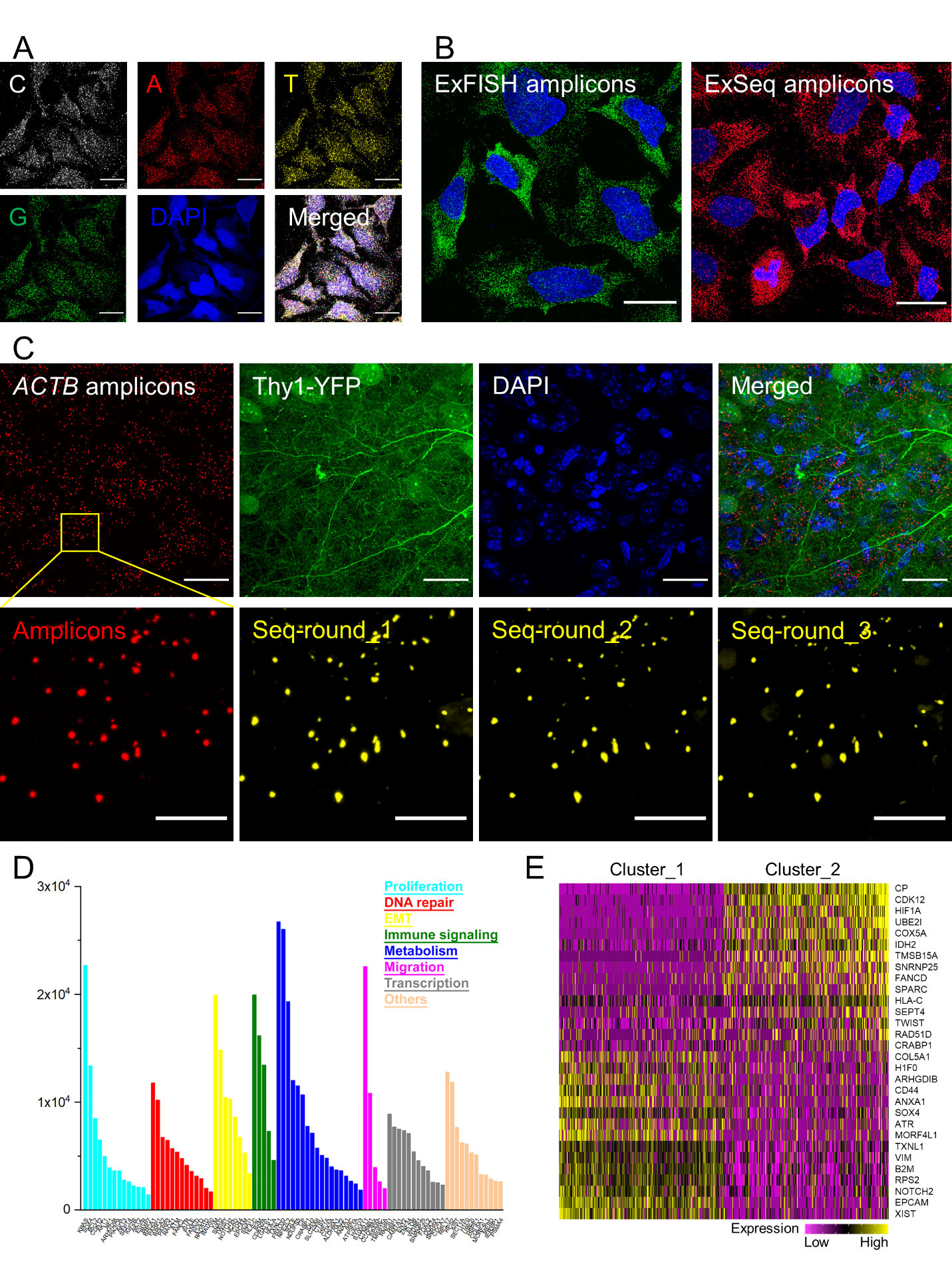


**S10 Fig.** **Demonstration of uniExM for *in situ* RNA sequencing (ExSeq). (A)** Amplicons generated by GMA-based uExSeq in HeLa cells were imaged with SBS reagents (from the Illumina MiSeq v3 kit). The following excitation and emission wavelengths were used for 5-channel acquisition: DAPI – Ex. 405 nm / Em. 440-460 nm; Base “G” – Ex. 488 nm / Em. 500-550 nm; Base “T” – Ex. 561 nm / Em. 575-590 nm; Base “A” – Ex. 640 nm / Em. 663-737 nm; Base “C” – Ex. 685 nm / Em. 705-845 nm. Scale bars (in pre-expansion units): 20 µm. **(B)** Characterization of uniExM for *in situ* enzymatic amplification in tExSeq. *GAPDH* mRNAs were amplified by HCR-FISH (ExFISH) or padlock probes in tExSeq. Then the generated signal spots in individual cells were counted. For better comparison, Alexa546 conjugated oligonucleotide probes were used for amplicon detection in both cases. Scale bars (in pre-expansion units): 20 µm. **(C)** Characterization of uniExM for *in situ* enzymatic sequencing in tExSeq. tExSeq targeting *ACTB* mRNAs in Thy1-YFP mouse brain tissues was performed, where padlock probes bearing consecutive bases “TTT” as the barcode were used. Before *in situ* sequencing, imaging with universal amplicon detection probes was performed to establish a reference image for the transcript locations (lower left, red). YFP signals were also imaged. After that, the universal probes and YFP signals were removed by concentrated formamide and heat treatment. Next, three rounds of SBS were conducted and the detected signal spots were benchmarked against the reference amplicon image (lower row, yellow dots). Scale bars (in pre-expansion units): 20 µm (for upper panel), 5 µm (for lower panel). **(D)** tExSeq targeting 87 cancer clone-specific genes in SA501 PDX breast cancer tissue was performed using 7-round SBS. All the decoded transcripts from a ~0.8 mm^2^ tissue slice, along with their function annotations, are summarized in the bar chart. **(E)** Principal component analysis (PCA) identified two groups of genes (15 each) that classify the tissue cells into two primary groups.


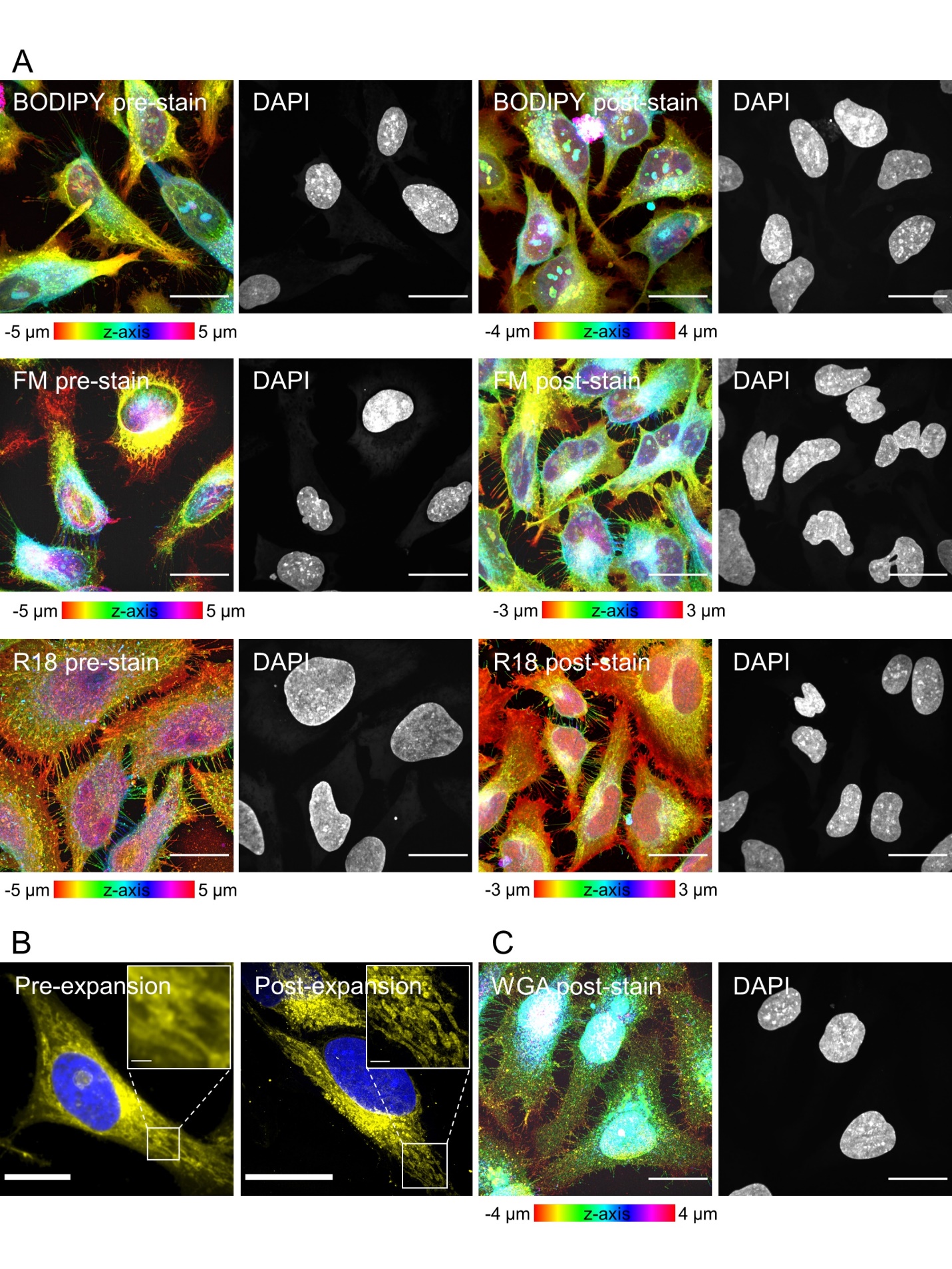


**S11 Fig.** **Qualitative demonstration of detection of lipids and carbohydrates in uniExM.** **(A)** Commercially available lipid staining reagents – BODIPY, FM and R18 – were tested in HeLa cells in the context of both pre- and post-expansion staining. As shown here, all three staining reagents exhibit consistent image patterns in pre- and post-expansion staining. The color-code in the lipid signal channel represents z-axis information. Scale bars (in pre-expansion units): 20 µm. **(B)** Detection of lipids expands the biological information that can be obtained by uniExM. In the expanded sample, post-expansion staining with lipophilic fluorophore R18 helps better resolve lipid-rich mitochondria where cristae are only discernible with expansion (highlighted in the zoomed-in inset). Scale bars (in pre-expansion units): 20 µm (large images); 1 µm (insets). **(C)** WGA-A647 was used to stain carbohydrates/glycoconjugates post-expansion in HeLa cells digested with the selective protease LysC. Strong signals were detected on cell and nuclear membranes. Color in the WGA channel represents z-axis information. Scale bars (in pre-expansion units): 20 µm.


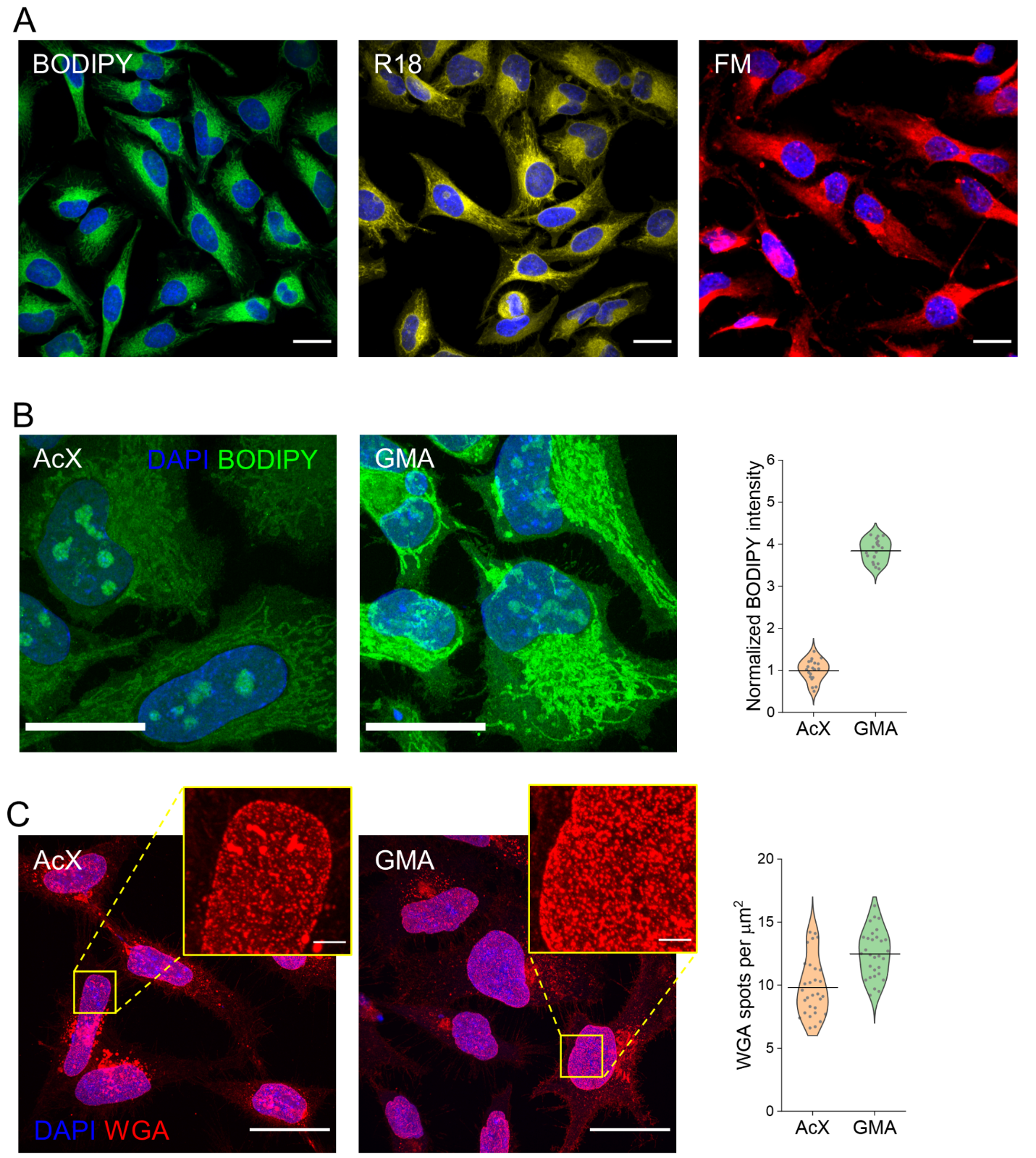


**S12 Fig.** **Qualitative and partial quantitative detection of lipids and glycoproteins (*i.e.,* carbohydrates) in HeLa cells treated with different anchors.** **(A)** Normal staining patterns for the three selected lipid tags in 4% PFA fixed HeLa cells. Scale bars: 20 µm. **(B)** Comparison between AcX and GMA treatments in visualizing lipid structures. HeLa cells were treated with 0.01% (w/v) AcX or 0.04% (w/v) GMA, followed by expansion and staining with DAPI and BODIPY. Fluorescence intensity measurements are presented in violin plots with mean values highlighted with solid lines. The intensity values were normalized to the level of AcX treated samples. (n = 20 images from 2 culture batches; two-sample *t*-test was performed for statistical significance, with *p* < 10^-10^). Scale bars (in pre-expansion units): 20 µm. **(C)** Comparison between AcX and GMA treatments in visualizing glycoprotein-enriched putative nucleoporins on nuclear membranes. Post-expansion staining with WGA in HeLa cells treated with GMA showed potentially better molecular retention, which in turn yielded a larger number of putative nucleoporin puncta, as compared to those of AcX treated samples. Scale bars (in pre-expansion units): 20 µm (zoomed-out images); 2 µm (zoomed-in images). WGA signal spots per µm^2^ were quantified (n = 30 cells from 2 culture batches; two-sample *t*-test was performed for statistical significance tests, with *p* < 10^-5^).


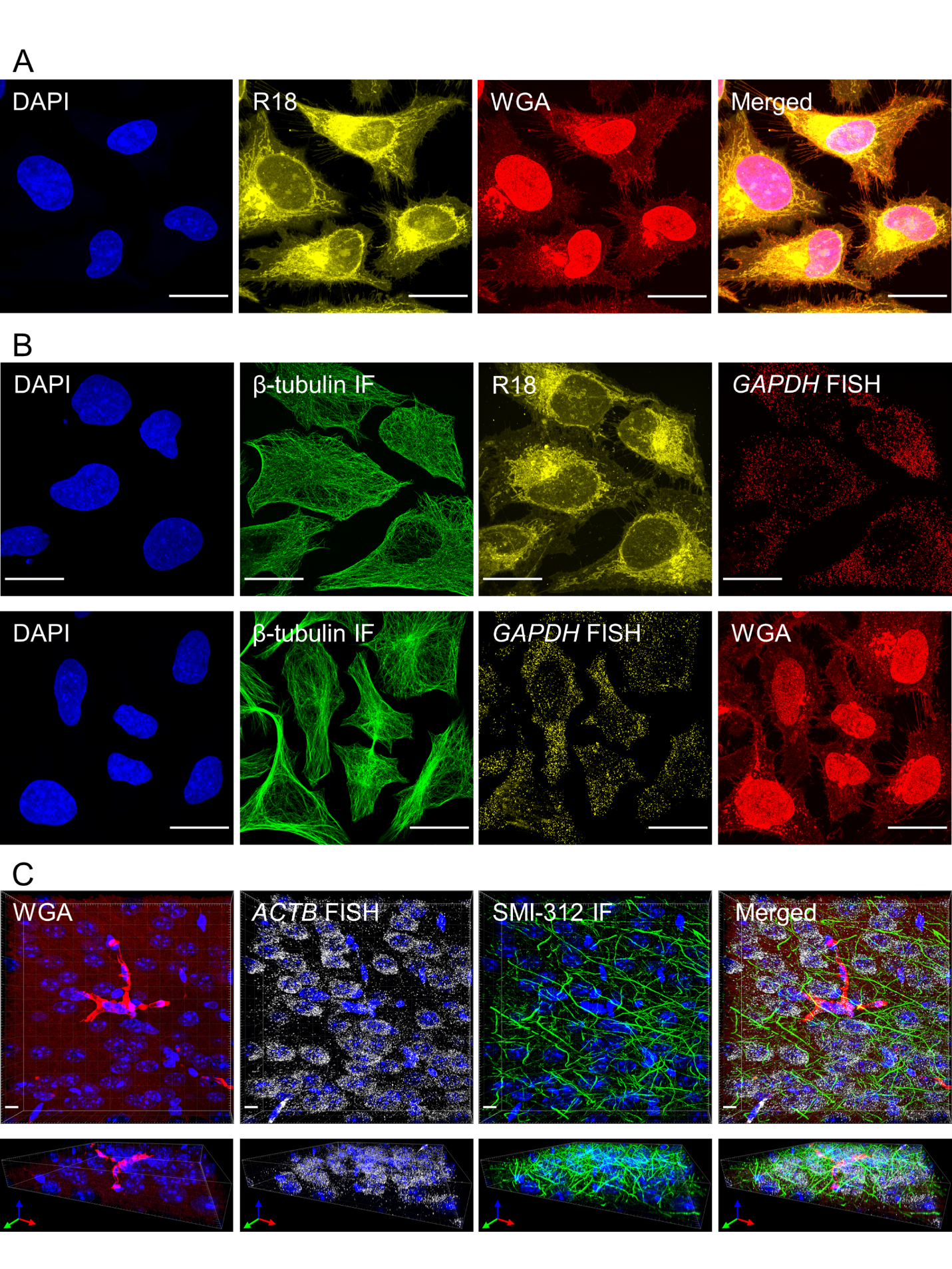


**S13 Fig.** **Qualitative demonstration of uniExM imaging of multiple biomolecular species. (A)** Demonstration of post-expansion co-staining for carbohydrates and lipids. HeLa cells were processed with 0.04% (w/v) GMA and LysC proteolysis, followed by staining with 10 µg/mL R18 and 5 µg/mL WGA-A647. Linear expansion factor: 4.3. Scale bars (in pre-expansion units): 20 µm. **(B)** Lipid or carbohydrate staining can be combined with protein and RNA detection in the same sample. As demonstrated in this figure, β-tubulin was stained with antibody pre-expansion, while R18, WGA staining and HCR-FISH were performed post-expansion. In addition to target-specific detection by IF (immunofluorescence) and FISH, staining for lipids and carbohydrates provides structural information at the cellular (*e.g.,* membranes) or subcellular levels (*e.g.,* mitochondria). Scale bars (in pre-expansion units): 20 µm. **(C)** Demonstration of multimodal detection by uniExM at the tissue level. In a 50 µm mouse brain tissue, HCR-FISH targeting *ACTB*, IF using SMI-312 antibody (against neurofilament) and WGA stain (contrast adjusted to highlight blood vessels) were applied together. Image stacks were rendered in 3D and presented in the lower panel. The colors in images correspond to the following fluorescent dyes: blue – DAPI; green – Alexa488; gray – Alexa546; red – Alexa647. Scale bars (in pre-expansion units): 5 µm.

**Supplementary Tables**


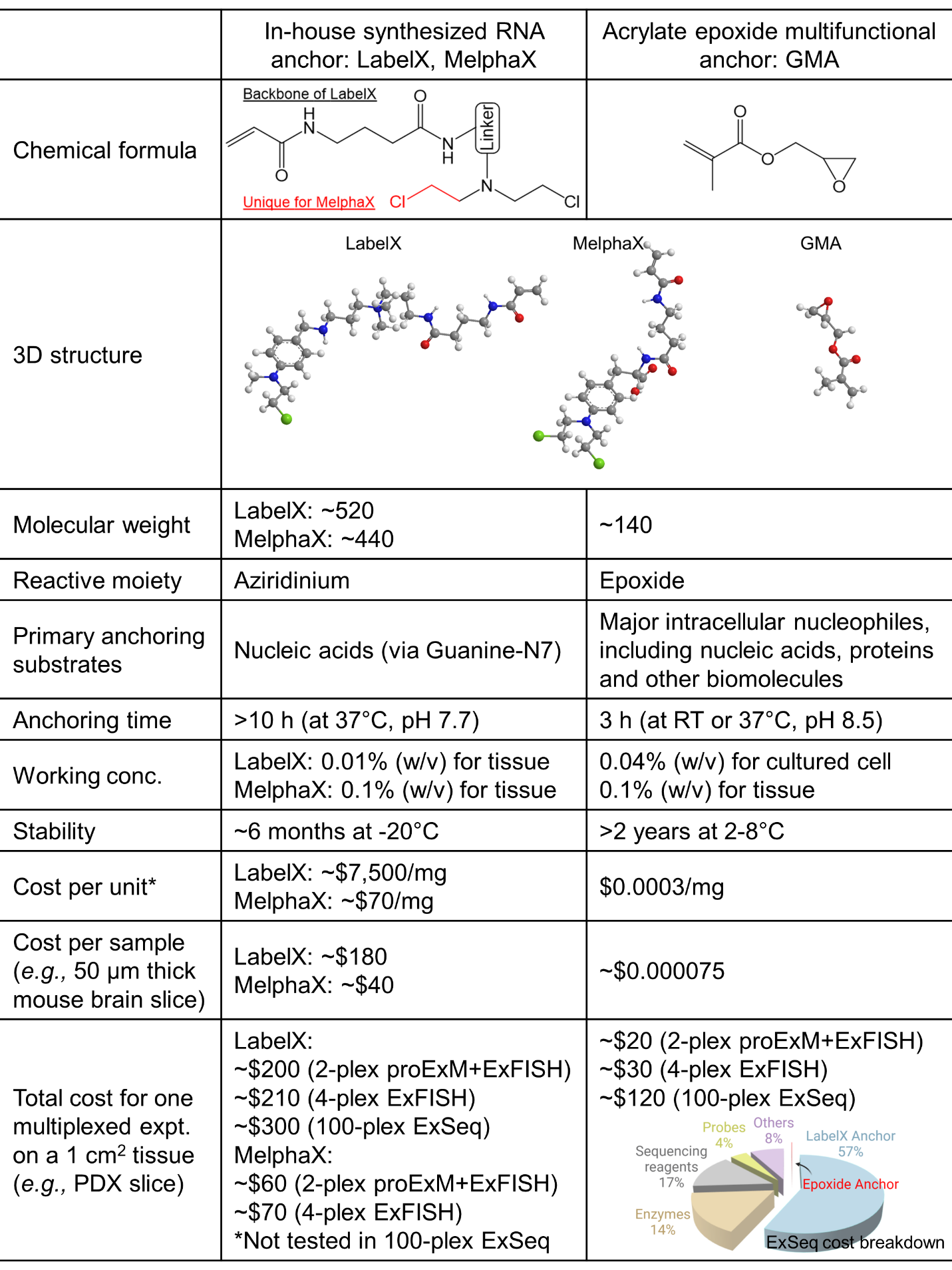


**S1 Table.** **High-level comparison of LabelX, MelphaX and GMA for key parameters in multimodal ExM applications**

| **Antibody name** | **Vendor information** | **Catalog number** |
| --- | --- | --- |
| Mouse anti-tubulin | DSHB | E7 |
| Rabbit anti-GFP | ThermoFisher Scientific | A-6455 |
| Mouse anti-βII-spectrin | BD Biosciences | 612563 |
| Mouse anti-MAP2 | BioLegend | 801801 |
| Mouse anti-SMI312 | BioLegend | 837904 |
| Goat anti-mouse IgG Alexa 488 | ThermoFisher Scientific | A-21430 |
| Goat anti-rabbit IgG Alexa 555 | ThermoFisher Scientific | A-11017 |
| Goat anti-mouse IgG Alexa 546 | ThermoFisher Scientific | A-11018 |
| Goat anti-rabbit IgG Alexa 647 | ThermoFisher Scientific | A-21246 |

**S2 Table.** **Main antibodies used in this study**

| **Chemical/Reagent Name** | **Supplier** | **Catalog #** |
| --- | --- | --- |
| 16% Formaldehyde (w/v), methanol-free (PFA) | Thermo Fisher | 28908 |
| 4′,6-diamidino-2-phenylindole (DAPI) | Sigma | D9542 |
| 4-Hydroxy-TEMPO (4-HT) | Sigma | 176141 |
| Acrylamide (AA) | Sigma | A9099 |
| Acrylamide/Bis 19:1, 40% (w/v) solution | Thermo Fisher | AM9022 |
| Acryloyl-X, SE, 6-((acryloyl)amino)hexanoic Acid, Succinimidyl Ester (AcX) | Thermo Fisher | A20770 |
| Aminoallyl-dUTP solution (50 mM) | Thermo Fisher | R1101 |
| Ammonium persulfate (APS) | Sigma | A3678 |
| Bind-silane | Sigma | GE17-1330-01 |
| BODIPY FL C_12_ | Thermo Fisher | D3822 |
| CircLigase II ssDNA ligase | Lucigen | CL9025K |
| Deoxynucleotide (dNTP), 10 mM solution mix | NEB | N0447L |
| Dideoxynucleoside triphosphate set (ddNTP) | Roche | 03732738001 |
| DMEM | Thermo Fisher | 10569010 |
| DMSO, Anhydrous | Thermo Fisher | D12345 |
| DTT | Roche | 37016821 |
| DPBS, 1× | Corning | 21-031-CV |
| EDTA, 0.5M solution, pH 8.0 | Thermo Fisher | 15575020 |
| Endonuclease V | NEB | M0305S |
| Endoproteinase LysC | NEB | P8109S |
| Ethanolamine hydrochloride | Sigma | E6133 |
| Fetal bovine serum | Thermo Fisher | 16000036 |
| FM 1-43 FX membrane stain | Thermo Fisher | F35355 |
| Formamide (deionized) | Thermo Fisher | AM9344 |
| Glutaraldehyde (GA), 25% solution | Sigma | G5882 |
| Glycidyl methylacrylate (GMA) | Sigma | 151238 |
| Guanidine hydrochloride | Sigma | 50937 |
| HCR-FISH probes, amplifiers and buffers | Molecular Instruments | N/A |
| Inosine | Sigma | I4125 |
| Label-IT amine | Mirus Bio | MIR3900 |
| MAXpack immunostaining media kit | Active Motif | 15251 |
| MES, 0.5M solution, pH 6.5 | Alfa Aesar | J63778 |
| MiSeq reagent kit v3 | Illumina | MS-102-3003 |
| N-(3-Dimethylaminopropyl)-N′-ethylcarbodiimide hydrochloride (EDC) | Sigma | 03450 |
| N,N,N′,N′-Tetramethylethylenediamine (TEMED) | Sigma | T7024 |
| N,N′-Methylenebisacrylamide (BIS) | Sigma | M7279 |
| N-Hydroxysuccinimide (NHS) | Thermo Fisher | 24500 |
| Octadecyl rhodamine B chloride (R18) | Thermo Fisher | O246 |
| PBCV-1 DNA ligase | NEB | M0375L |
| PBS, 10× | Thermo Fisher | 70011044 |
| PEGylated bis(sulfosuccinimidyl)suberate (BE(PEG)9) | Thermo Fisher | 21582 |
| Penicillin-streptomycin (10,000 U/mL) | Thermo Fisher | 15140122 |
| phi29 DNA polymerase | Enzymatics | P7020-HC-L |
| Proteinase K | NEB | P8107S |
| RNase A | Thermo Fisher | EN0531 |
| RNase H | NEB | M0297L |
| RNase inhibitor | NEB | M0314S |
| Sigmacote | Sigma | SL2 |
| Sodium acrylate (SA) | Sigma | 408220 |
| Sodium bicarbonate, powder | Sigma | S6014 |
| Sodium borate, 0.5M solution, pH 8.5 | Alfa Aesar | J62902 |
| Sodium borohydride | Sigma | 213462 |
| Sodium chloride, 5M solution | Sigma | 59222C |
| Sodium dodecyl sulfate (SDS), 20% solution | Sigma | 05030 |
| SSC buffer, 20× | Promega | V4261 |
| Standard Taq reaction buffer (with magnesium chloride) | NEB | B9014S |
| SuperScript IV reverse transcriptase | Thermo Fisher | 18090050 |
| Terminal transferase | NEB | M0315L |
| TetraSpeck microspheres, 0.5 µm | Thermo Fisher | T7281 |
| Trimethylolpropane triglycidyl ether (TMPTE) | Sigma | 430269 |
| Tris buffer, 1M solution, pH 8.0, RNase-free | Thermo Fisher | AM9856 |
| Triton X-100 | Sigma | T8787 |
| Trypsin-EDTA (0.25% with phenol red) | Thermo Fisher | 25200072 |
| Tween 20 | Sigma | P9416 |
| UltraPure DNase/RNase-free distilled water | Thermo Fisher | 10977023 |
| Wheat germ agglutinin (WGA)-Alexa Fluor 647 | Thermo Fisher | W32466 |
| Zwittergent 3-10 detergent | Sigma | 693021 |

**S3 Table.** **Main chemicals and reagents used in this study**

| **Figure** | **Sample type** | **Anchoring condition** | **Detection module** |
| --- | --- | --- | --- |
| Fig. 1B (i-ii) | Mouse brain slices | 0.1% GMA, 100 mM NaHCO_3_ (pH 8.5), 3-6 h pre-incubation at 4°C and 3 h at 37°C | post-expansion HCR-FISH +  YFP signal analysis |
| Fig. 1B (iii) | Human HeLa cells;  Mouse hippocampal neurons | 0.04% GMA, 100 mM NaHCO_3_ (pH 8.5), 3 h at 37°C | pre-expansion IF +  post-expansion HCR-FISH |
| Fig. 2A | Human HeLa cells | 0.04% GMA, 100 mM NaHCO_3_ (pH 8.5), 3 h at RT | pre-expansion IF |
| Fig. 2B | Human HeLa cells | 0.04% GMA, 100 mM NaHCO_3_ (pH 8.5), 3 h at RT | pre-expansion HCR-FISH +  post-expansion HCR-FISH |
| Fig. 3 | Mouse hippocampal neurons | 0.04% GMA, 100 mM NaHCO_3_ (pH 8.5), 3 h at 37°C | pre- or post-expansion IF |
| Fig. 4B (ii) Fig. S10A | Human HeLa cells | 0.04% GMA, 100 mM NaHCO_3_ (pH 8.5), 6 h at RT | untargeted in situ sequencing |
| Fig. 4C | Human PDX breast cancer tissues | 0.1% GMA, 6 h at 4°C (with 1× PBS, pH 7.4) plus overnight at RT (with 100 mM NaHCO_3_, pH 8.5) | targeted in situ sequencing |
| Fig. S3 | Human HeLa cells;  Mouse brain slices | 0.04% GMA, 100 mM NaHCO_3_ (pH 8.5), 3 h at RT; 0.1% GMA, 100 mM NaHCO_3_ (pH 8.5), 3-6 h pre-incubation at 4°C and 3 h at 37°C (for mouse brain) | post-expansion IF or post-expansion HCR-FISH |
| Fig. S4 | Human HeLa cells | 0.04% GMA, 100 mM NaHCO_3_ (pH 8.5), 3 h at RT | DAPI staining before imaging |
| Fig. S5 | Human HeLa cells | as indicated in plots | post-expansion HCR-FISH |
| Fig. S6 | Human HeLa cells | 0.04% GMA, 100 mM NaHCO_3_ (pH 8.5), 3 h at RT | post-expansion HCR-FISH |
| Fig. S7 | Mouse hippocampal neurons | 0.04% GMA, 100 mM NaHCO_3_ (pH 8.5), 3 h at 37°C | pre- or post-expansion IF |
| Fig. S8 | Mouse brain slices | 0.05% GMA + 0.05% TMPTE, 6 h at 4°C (with 1× PBS, pH 7.4) plus overnight at RT (with 100 mM NaHCO_3_, pH 8.5) | post-expansion YFP signal analysis |
| Fig. S10C | Mouse brain slices | 0.1% GMA, 6 h at 4°C (with 1× PBS, pH 7.4) plus overnight at RT (with 100 mM NaHCO_3_, pH 8.5) | targeted in situ sequencing |
| Fig. S11A-B  Fig. S12B | Human HeLa cells | 0.04% GMA, overnight at 4°C (with 1× PBS, pH 7.4) plus 3 h at RT (with 100 mM NaHCO_3_, pH 8.5) | pre-expansion or post-expansion lipid tag staining |
| Fig. S11C  Fig. S12C | Human HeLa cells | 0.04% GMA, 100 mM NaHCO_3_ (pH 8.5), 3 h at RT | post-expansion WGA staining |
| Fig. S13A | Human HeLa cells | 0.04% GMA, overnight at 4°C (with 1× PBS, pH 7.4) plus 3 h at RT (with 100 mM NaHCO_3_, pH 8.5) | post-expansion R18 + WGA staining |
| Fig. S13B | Human HeLa cells | 0.04% GMA, overnight at 4°C (with 1× PBS, pH 7.4) plus 3 h at RT (with 100 mM NaHCO_3_, pH 8.5) | pre-expansion IF +  post-expansion HCR-FISH + post-expansion R18 or WGA staining |
| Fig. S13C | Mouse brain slices | 0.1% GMA, overnight at 4°C (with 1× PBS, pH 7.4) plus 3 h at RT (with 100 mM NaHCO3, pH 8.5) | pre-expansion IF +  post-expansion HCR-FISH + post-expansion WGA staining |

**S4 Table.** **Anchoring conditions used in main experiments**

| **Gene function annotation** | | | | | | | |
| --- | --- | --- | --- | --- | --- | --- | --- |
| **Proliferation** | **DNA repair** | **EMT** | **Immune signaling** | **Metabolism** | **Migration** | **Transcription** | **Others** |
| KRAS  SOX4  BCL2  CDK12  AKT1  NF1  ARHGDIB  PIK3CA  AKT3  SEPT4  IGF2R  AKT2  EGFR  BMP7 | BRCA1  PARP1  BRCA2  PARP3  RAD21  ATM  FANCL  ATR  POLE  FANCD  POLQ  RAD51D  RAD50 | VIM  SNAI2  TWIST  NOTCH2  NCAD  CD44  EPCAM  SNAI1 | HLA-C  B2M  CDKN2A  LGALS1  HLA-A | TXNL1  FBXO32  NFE2L2  SQLE  NDUFS5  CP  CRABP1  IDH2  SLC25A6  CTSV  HIF1A  COX5A  ALDH9A1  OAZ2  ANXA1  ARC  ATP5F1A  HACD3 | S100A11  CTNNB1  CCDC88A  SPARC  TMSB15A | RNPS1  RBP1  CAMTA1  ENY2  ZNF24  ELOB  WDR61  SNRNP25  FOXL2  DDX24  SNRPA1  CDCA7 | RPL17  RPS2  XIST  SEC11A  H1F0  UBE2I  HSPE1  COL5A1  MORF4L1  MESD  IER3IP1  PSMA4 |

**S5 Table.** **Gene list used for profiling SA501 PDX cancer tissues**
